# Supplementary material for: Sociodemographic differences in self-reported exposure to high fat, salt and sugar food and drink advertising: a cross-sectional analysis of 2019 UK panel data
Source: BMJ Open. 2021 Apr 7;11(4):e048139. doi: 10.1136/bmjopen-2020-048139 (PMC8031692; doi:10.1136/bmjopen-2020-048139)
Supplement: Supplementary data [file bmjopen-2020-048139supp001.pdf]

SUPPLEMENTARY MATERIAL

Supplementary Table S1. Definitions of product categories as presented in survey questions

Product category	Definition in survey
Processed HFSS foods	Processed foods high in salt, sugar and fat are those such as sugary drinks, meals from fast food chains, ready meals, sit down meals, sugary breakfast cereals, sweet snacks (e.g. chocolate bars, sweets, cookies/biscuits), savoury snacks (e.g. crisps, salted/flavoured nuts) and desserts (cakes, ice-cream and flavoured yoghurts).
Sugary drinks	SUGARY DRINKS are drinks that contain added sugar, like fizzy drinks, fruit drinks, sports drinks, energy drinks, diluted cordials/squash, chocolate milk, and specialty flavoured hot drinks that have added sugar but DO NOT INCLUDE pure fruit juice.
Sugary cereals	SUGARY CEREALS are those that contain added sugar such as chocolate or other flavouring/ fillings e.g. Coco Pops, Krave or are sweetened with frosting or added sugar e.g. Frosties, Sugar Puffs. DO NOT INCLUDE cereals like Weetabix, Ready Brek, Oatmeal/Porridge).
Sweet snacks	SWEET SNACKS are foods such as chocolate bars, cookies/biscuits, danish pastries, granola bars, sweets or other confectionary.
Fast food	These can be MEALS, FOODS OR DRINKS from fast-food chains or take-aways (e.g., Burger King, Dominos, McDonalds, KFC, fish and chip shops, Indian or Chinese Takeaway).

Supplementary Table S2. Number (%) of participants who reported seeing or hearing advertising for less healthy products and digital food delivery services in the past 7 days by sociodemographic characteristics, stratified by HFSS product category (*n*=1552)

Sociodemographic characteristic		Any	Processed HFSS foods	Sugary drinks	Sugary cereals	Sweet snacks	Fast food	Digital food delivery services
All		1314 (84.7)	1059 (68.2)	813 (52.4)	653 (42.1)	853 (55.0)	1107 (71.3)	852 (54.9)
Sex, N (%)	Male	370 (83.9)	277 (62.8)	228 (51.7)	195 (44.2)	235 (53.3)	316 (71.7)	249 (56.5)
	Female	944 (85.0)	782 (70.4)	585 (52.7)	458 (41.2)	618 (55.6)	791 (71.2)	603 (54.3)
X^2 (P-value)		0.28 (0.598)	8.36 (0.004)**	0.11 (0.734)	1.16 (0.281)	0.70 (0.404)	0.03 (0.857)	0.61 (0.435)
Age group (years), N (%)	18-34	157 (83.5)	129 (68.6)	96 (51.1)	74 (36.4)	105 (55.9)	131 (69.7)	113 (60.1)
	35-44	253 (84.6)	211 (70.6)	155(51.8)	134 (44.8)	161 (53.9)	210 (70.2)	176 (58.9)
	45-54	348 (84.7)	263 (64.0)	206 (50.1)	166 (40.4)	216 (52.6)	294 (71.5)	220 (53.5)
	55-64	286 (85.4)	235 (70.2)	190 (56.7)	151 (45.1)	193 (57.6)	252 (75.2)	191 (57.0)
	≥65	270 (84.6)	221 (69.3)	166 (52.0)	128 (40.1)	178 (55.8)	220 (69.0)	152 (47.7)
X^2 (P-value)		0.32 (0.988)	4.91 (0.297)	3.55 (0.471)	3.70 (0.448)	2.21 (0.697)	3.79 (0.435)	11.6 (0.020)*
Socioeconomic position, N (%)	AB	276 (80.9)	210 (61.6)	165 (48.4)	119 (34.9)	169 (49.6)	231 (67.7)	168 (49.3)
	C1C2	798 (86.2)	643 (69.4)	492 (53.1)	410 (44.3)	523 (56.5)	671 (72.5)	525 (56.7)
	DE	240 (84.2)	206 (72.3)	156 (54.7)	124 (43.5)	161 (56.5)	205 (71.9)	159 (55.8)
X^2 (P-value)		5.32 (0.070)	9.73 (0.008)*	3.02 (0.220)	9.29 (0.010)*	5.15 (0.076)	2.78 (0.249)	5.67 (0.059)
Children in the household, N (%)	No	951 (85.7)	768 (69.2)	598 (53.9)	465 (41.9)	621 (56.0)	807 (72.7)	615 (55.4)
	Yes	363 (82.1)	291 (65.8)	215 (48.6)	188 (42.5)	232 (52.5)	300 (67.9)	237 (53.6)
X^2 (P-value)		3.07 (0.080)	1.64 (0.200)	3.47 (0.063)	0.05 (0.817)	1.53 (0.217)	3.61 (0.058)	0.41 (0.523)
Working status, N (%)	Full-time	519 (84.8)	418 (68.3)	328 (53.6)	267 (43.6)	350 (57.2)	445 (72.7)	365 (59.6)
	Part-time	187 (83.9)	151 (67.7)	112 (50.2)	91 (40.8)	116 (52.0)	154 (69.1)	115 (51.6)
	Self-employed	113 (86.3)	82 (62.6)	64 (48.9)	49 (37.4)	68 (51.9)	97 (74.1)	67 (51.2)
	Retired	290 (84.8)	241 (70.5)	181 (52.9)	145 (42.4)	192 (56.1)	241 (70.5)	171 (50.0)
	Not looking/ unable to work	179 (83.6)	148 (69.2)	111 (51.9)	87 (40.7)	110 (51.4)	151 (70.6)	117 (54.7)
	Other	26 (86.7)	19 (63.3)	17 (56.7)	14 (46.7)	17 (56.7)	19 (63.3)	17 (56.7)
X^2 (P-value)		0.65 (0.986)	3.15 (0.676)	1.71 (0.887)	2.38 (0.795)	3.82 (0.575)	2.73 (0.741)	10.66 (0.059)
Region, N (%)	London	706 (83.4)	485 (68.8)	390 (55.3)	300 (42.6)	377 (53.5)	509 (72.2)	416 (59.0)
	North	608 (86.2)	574 (67.8)	423 (49.9)	353 (41.7)	476 (56.2)	598 (70.6)	436 (51.5)
X^2 (P-value)		2.47 (0.116)	0.19 (0.666)	4.46 (0.035)*	0.12 (0.728)	1.15 (0.283)	0.48 (0.489)	8.81 (0.003)**
* P<0.05, ** P<0.01, *** P<0.001								

Supplementary Table S3. Number (%) of participants who reported seeing or hearing advertising for less healthy products and digital food delivery services in the past 7 days by sociodemographic characteristics, stratified by advertising setting (*n*=1552)

**Sociodemographic characteristic**		**Traditional**	**Digital**	**Recreational**	**Functional**	**Transport**
All		1148 (74.0)	600 (38.7)	292 (18.8)	799 (51.5)	565 (36.4)
Sex, N (%)	Male	318 (72.1)	179 (40.6)	87 (19.7)	231 (52.4)	172 (39.0)
	Female	830 (74.7)	421 (37.9)	205 (18.5)	568 (51.1)	393 (35.4)
X^2 (P-value)		1.11 (0.293)	0.97 (0.325)	0.34 (0.562)	0.20 (0.655)	1.80 (0.180)
Age group (years), N (%)	18-34	130 (69.2)	108 (57.5)	41 (21.8)	107 (56.9)	87 (46.3)
	35-44	215 (71.9)	155 (51.8)	61 (20.4)	161 (53.9)	128 (42.8)
	45-54	300 (73.0)	157 (38.2)	66 (16.1)	204 (49.6)	144 (35.0)
	55-64	258 (77.0)	109 (32.5)	69 (20.6)	173 (51.6)	109 (32.5)
	≥65	245 (76.8)	71 (22.3)	55 (17.2)	154 (48.3)	97 (30.4)
X^2 (P-value)		6.08 (0.193)	91.4 (<0.001)***	4.85 (0.303)	4.77 (0.312)	20.66 (<0.001)***
Socioeconomic position, N (%)	AB	231 (67.7)	121 (35.5)	64 (18.8)	167 (49.0)	125 (36.7)
	C1C2	703 (75.9)	363 (39.2)	175 (18.9)	484 (52.3)	342 (36.9)
	DE	214 (75.1)	116 (40.7)	53 (18.6)	148 (51.9)	98 (34.4)
X^2 (P-value)		8.88 (0.012)**	2.07 (0.356)	0.01 (0.993)	1.11 (0.574)	0.62 (0.732)
Children in the household, N (%)	No	834 (75.1)	391 (35.2)	217 (19.6)	573 (51.6)	401 (36.1)
	Yes	314 (71.0)	209 (47.3)	75 (17.0)	226 (51.1)	164 (37.1)
X^2 (P-value)		2.75 (0.097)	19.39 (<0.001)***	1.38 (0.240)	0.03 (0.862)	0.13 (0.718)
Working status, N (%)	Full-time	451 (73.7)	284 (46.4)	126 (20.6)	333 (54.4)	254 (41.5)
	Part-time	161 (72.2)	91 (40.8)	40 (17.9)	107 (48.0)	72 (32.3)
	Self-employed	93 (71.0)	50 (38.2)	21 (16.0)	68 (51.9)	57 (43.5)
	Retired	262 (76.6)	78 (22.8)	59 (17.3)	163 (47.7)	100 (29.2)
	Not looking/ unable to work	160 (74.8)	88 (41.1)	38 (17.8)	113 (52.8)	66 (30.8)
	Other	21 (70.0)	9 (30.0)	8 (26.7)	15 (50.0)	16 (53.3)
X^2 (P-value)		2.54 (0.770)	53.67 (<0.001)***	3.95 (0.556)	5.38 (0.371)	25.52 (<0.001)***
Region, N (%)	London	528 (75.9)	287 (40.7)	139 (19.7)	376 (53.3)	321 (45.5)
	North	620 (73.2)	313 (37.0)	153 (18.1)	423 (49.9)	244 (28.8)
X^2 (P-value)		0.57 (0.449)	2.29 (0.130)	0.69 (0.407)	1.77 (0.183)	46.48 (<0.001)***
* P<0.05, ** P<0.01, *** P<0.001						

Supplementary Table S4. Number (%) of participants who reported seeing or hearing advertising for less healthy products and digital food delivery services in the past 7 days by product/service advertised and advertising setting, stratified by region (*n*=1552)

**Advertised product/service**	**Region**	All	Traditional	Digital	Recreational	Functional	Transport
All, N (%)	All	1314 (84.7)	1148 (74.0)	600 (38.7)	292 (18.8)	799 (51.5)	565 (36.4)
	London	608 (86.2)	528 (74.9)	287 (40.7)	139 (19.7)	376 (53.3)	321 (45.5)
	North	706 (83.4)	620 (73.2)	313 (37.0)	153 (18.1)	423 (49.9)	244 (28.8)
X^2 (P-value)		2.47 (0.116)	0.57 (0.449)	2.29 (0.130)	0.69 (0.407)	1.77 (0.183)	46.48 (<0.001)***
Processed HFSS foods, N (%)	All	1059 (68.2)	853 (55.0)	384 (24.7)	135 (8.7)	547 (35.2)	376 (24.2)
	London	485 (68.8)	387 (54.9)	177 (25.1)	59 (8.4)	253 (35.9)	223 (31.6)
	North	574 (67.8)	466 (55.0)	207 (24.4)	76 (9.0)	294 (34.7)	153 (18.1)
X^2 (P-value)		0.19 (0.666)	0.002 (0.961)	0.09 (0.762)	0.18 (0.674)	0.23 (0.629)	38.58 (<0.001)***
Sugary drinks, N (%)	All	813 (52.4)	596 (38.3)	223 (14.4)	114 (7.4)	318 (20.5)	206 (13.3)
	London	390 (55.3)	279 (39.6)	105 (14.9)	56 (7.9)	155 (22.0)	117 (16.6)
	North	423 (49.9)	316 (37.3)	118 (13.9)	58 (6.9)	163 (19.2)	89 (10.5)
X^2 (P-value)		4.46 (0.035)*	0.84 (0.361)	0.29 (0.591)	0.68 (0.410)	1.77 (0.183)	12.39 (<0.001)***
Sugary cereals, N (%)	All	653 (42.1)	502 (32.4)	123 (7.9)	48 (3.1)	215 (13.9)	75 (4.8)
	London	300 (42.6)	230 (32.6)	62 (8.8)	26 (3.7)	100 (14.2)	56 (7.9)
	North	353 (41.7)	272 (32.1)	61 (7.2)	22 (2.6)	115 (13.6)	19 (2.2)
X^2 (P-value)		0.12 (0.728)	0.05 (0.830)	1.33 (0.248)	1.53 (0.217)	0.12 (0.730)	27.18 (<0.001)***
Sweet snacks, N (%)	All	853 (55.0)	639 (41.2)	218 (14.1)	101 (6.5)	339 (21.8)	171 (11.0)
	London	377 (53.5)	273 (38.7)	101 (14.3)	45 (6.4)	163 (23.1)	109 (15.5)
	North	476 (56.2)	366 (43.2)	117 (13.8)	56 (6.6)	176 (20.8)	62 (7.3)
X^2 (P-value)		1.15 (0.283)	3.20 (0.074)	0.08 (0.772)	0.03 (0.856)	1.24 (0.266)	26.01 (<0.001)***
Fast food, N (%)	All	1107 (71.3)	855 (55.1)	312 (20.1)	103 (6.6)	380 (24.5)	327 (21.1)
	London	509 (72.2)	386 (54.8)	137 (19.4)	53 (7.5)	179 (25.4)	198 (28.1)
	North	598 (70.6)	469 (55.4)	175 (20.7)	50 (5.9)	201 (23.7)	129 (15.2)
X^2 (P-value)		0.48 (0.489)	0.06 (0.807)	0.36 (0.548)	1.62 (0.203)	0.57 (0.449)	38.23 (<0.001)***
Digital food delivery services, N (%)	All	852 (54.9)	647 (41.7)	297 (19.1)	37 (2.4)	115 (7.4)	126 (8.1)
	London	416 (59.0)	299 (42.4)	163 (23.1)	34 (3.4)	66 (9.4)	96 (13.6)
	North	436 (51.5)	348 (41.1)	134 (15.8)	13 (1.5)	49 (5.8)	30 (3.5)
X^2 (P-value)		8.81 (0.003)**	0.28 (0.598)	13.25 (<0.001)**	5.78 (0.016)*	7.17 (0.007)*	52.36 (<0.001)***
* P<0.05, ** P<0.01, *** P<0.001							

Supplementary Table S5. Interaction terms (P-values) for interactions between sociodemographic characteristics and region on self-reported advertising exposure

**Advertising type**	**Sex##Region**	**Age group##Region**	**Socioeconomic position##Region**	**Children##Region**	**Working status##Region**
Any advertising	1.52 (0.677)	3.91 (0.917)	5.81 (0.325)	4.23 (0.238)	2.83 (0.993)
Processed HFSS foods	10.64 (0.014)**	7.35 (0.600)	9.64 (0.086)	3.41 (0.332)	6.15 (0.863)
Sugary drinks	2.31 (0.511)	6.40 (0.700)	6.62 (0.251)	3.45 (0.327)	4.59 (0.949)
Sugary cereal	1.96 (0.580)	5.76 (0.764)	13.08 (0.023)*	2.32 (0.508)	14.30 (0.217)
Sweet snacks	2.60 (0.457)	3.83 (0.922)	8.88 (0.114)	1.47 (0.690)	9.19 (0.605)
Fast food	0.08 (0.995)	4.05 (0.908)	3.37 (0.643)	3.48 (0.323)	4.28 (0.961)
Digital food delivery services	4.27 (0.233)	16.41 (0.059)	8.56 (0.128)	4.15 (0.245)	13.14 (0.284)
Traditional advertising	2.26 (0.520)	7.82 (0.553)	11.37 (0.044)*	1.39 (0.709)	9.29 (0.595)
Digital advertising	5.79 (0.122)	47.08 (<0.001)***	9.82 (0.081)	3.27 (0.352)	22.98 (0.018)*
Advertising in recreational environments	2.32 (0.508)	10.03 (0.348)	3.90 (0.564)	5.17 (0.160)	5.90 (0.880)
Advertising in functional environments	1.60 (0.660)	9.93 (0.357)	3.81 (0.577)	3.20 (0.362)	9.51 (0.575)
Advertising across transport networks	5.31 (0.151)	29.90 (<0.001)***	7.43 (0.190)	7.25 (0.065)	16.69 (0.117)

* P<0.05, ** P<0.01, *** P<0.001

Supplementary Table S6. Sociodemographic correlates of advertising exposure stratified by product/service advertised and region where interactions were significant (*n*=1552)

Product/service advertised	Sex	Age group	Socioeconomic position	Children in the household	Working status
Any advertising	No interaction	No interaction	No interaction	No interaction	No interaction
Processed HFSS foods	London<br>1.18 (0.82-1.70)<br>North of England<br>1.70 (1.22-2.37)**	No interaction	No interaction	No interaction	No interaction
Sugary drinks	No interaction	No interaction	No interaction	No interaction	No interaction
Sugary cereal	No interaction	No interaction	London<br>C1C2: 1.35 (0.94-1.95)<br>DE: 1.75 (1.06-2.88)*<br>North of England<br>C1C2: 1.62 (1.11-2.36)*<br>DE: 1.41 (0.88-2.25)	No interaction	No interaction
Sweet snacks	No interaction	No interaction	No interaction	No interaction	No interaction
Fast food	No interaction	No interaction	No interaction	No interaction	No interaction
Digital food delivery services	No interaction	No interaction	No interaction	No interaction	No interaction

Supplementary Table S7. Sociodemographic correlates of advertising exposure stratified by advertising setting and region where interactions were significant (*n*=1552)

Advertising setting	Sex	Age group	Socioeconomic position	Children in the household	Working status
Traditional	No interaction	No interaction	London<br>C1C2: 1.41 (0.95-2.10)<br>DE: 1.90 (1.06-3.41)*<br>North of England<br>C1C2: 1.53 (1.04-2.25)*<br>DE: 1.17 (0.72-1.91)	No interaction	No interaction
Digital	No interaction	London<br>35-44: 0.84 (0.45-1.55)<br>45-54: 0.37 (0.20-0.68)**<br>55-64: 0.27 (0.14-0.52)***<br>≥65: 0.15 (0.06-0.37)***<br>North of England<br>35-44: 0.72 (0.44-1.18)<br>45-54: 0.46 (0.28-0.74)**<br>55-64: 0.42 (0.25-0.71)***<br>≥65: 0.37 (0.17-0.82)***	No interaction	No interaction	London<br>Part-time: 0.69 (0.40-1.16)<br>Self-employed: 0.69 (0.40-1.17)<br>Retired: 0.64 (0.31-1.35)<br>At home: 0.50 (0.29-0.88)*<br>Other: 0.08 (0.02-0.37)**<br>North of England<br>Part-time: 0.99 (0.64-1.55)<br>Self-employed: 0.71 (0.38-1.33)<br>Retired: 0.67 (0.34-1.32)<br>At home: 1.06 (0.67-1.66)<br>Other: 2.67 (0.75-9.55)
Recreational	No interaction	No interaction	No interaction	No interaction	No interaction
Functional	No interaction	No interaction	No interaction	No interaction	No interaction
Transport	No interaction	London<br>35-44: 0.72 (0.39-1.30)<br>45-54: 0.52 (0.29-0.93)*<br>55-64: 0.55 (0.29-1.04)*<br>≥65: 0.73 (0.32-1.68)<br>North of England<br>35-44: 0.86 (0.52-1.43)<br>45-54: 0.49 (0.30-0.82)**<br>55-64: 0.44 (0.25-0.77)**<br>≥65: 0.34 (0.15-0.78)**	No interaction	No interaction	No interaction
