# Supplementary material for: Sociodemographic differences in self-reported exposure to high fat, salt and sugar food and drink advertising: a cross-sectional analysis of 2019 UK panel data
Source: BMJ Open. 2021 Apr 7;11(4):e048139. doi: 10.1136/bmjopen-2020-048139 (PMC8031692; doi:10.1136/bmjopen-2020-048139)
Supplement: Author's manuscript [file bmjopen-2020-048139.draft_revisions.pdf]

BMJ Open

BMJ Open is committed to open peer review. As part of this commitment we make the peer review history of every article we publish publicly available.

When an article is published we post the peer reviewers' comments and the authors' responses online. We also post the versions of the paper that were used during peer review. These are the versions that the peer review comments apply to.

The versions of the paper that follow are the versions that were submitted during the peer review process. They are not the versions of record or the final published versions. They should not be cited or distributed as the published version of this manuscript.

BMJ Open is an open access journal and the full, final, typeset and author-corrected version of record of the manuscript is available on our site with no access controls, subscription charges or pay-per-view fees (http://bmjopen.bmj.com).

If you have any questions on BMJ Open's open peer review process please email
info.bmjopen@bmj.com

BMJ Open

Sociodemographic differences in self-reported exposure to less healthy food and drink advertising: a cross-sectional analysis of UK panel data

Journal:	*BMJ Open*
Manuscript ID	bmjopen-2020-048139
Article Type:	Original research
Date Submitted by the Author:	04-Jan-2021

Complete List of Authors:	Yau, Amy; London School of Hygiene & Tropical Medicine, Public Health, Environments & Society<br>Adams, J; University of Cambridge, Centre for Diet & Activity Research<br>Boyland, Emma; University of Liverpool, Department of Psychology<br>Burgoine, Thomas; University of Cambridge, Centre for Diet and Activity Research<br>De Vocht, Frank; University of Bristol, Population Health Sciences; National Institute for Health Research Applied Research Collaboration West<br>Egan, Matt; London School of Hygiene & Tropical Medicine, Public Health, Environments & Society<br>Lake, Amelia; Teesside University, Centre for Public Health Research; Centre for Translational Research in Public Health, Fuse<br>Lock, Karen; London School of Hygiene & Tropical Medicine, Health Services Research & Policy<br>Mytton, Oliver; University of Cambridge, Centre for Diet and Activity Research<br>Petticrew, Mark; London School of Hygiene & Tropical Medicine, Public Health, Environments & Society<br>Thompson, Claire; University of Hertfordshire, Centre for Research in Public Health and Community Care<br>White, Martin; University of Cambridge, Centre for Diet and Activity Research<br>Cummins, Steven; London School of Hygiene & Tropical Medicine, Public Health, Environments & Society

Keywords:	PUBLIC HEALTH, SOCIAL MEDICINE, NUTRITION & DIETETICS

SCHOLARONE™
Manuscripts

I, the Submitting Author has the right to grant and does grant on behalf of all authors of the Work (as defined in the below author licence), an exclusive licence and/or a non-exclusive licence for contributions from authors who are: i) UK Crown employees; ii) where BMJ has agreed a CC-BY licence shall apply, and/or iii) in accordance with the terms applicable for US Federal Government officers or employees acting as part of their official duties; on a worldwide, perpetual, irrevocable, royalty-free basis to BMJ Publishing Group Ltd ("BMJ") its licensees and where the relevant Journal is co-owned by BMJ to the co-owners of the Journal, to publish the Work in this journal and any other BMJ products and to exploit all rights, as set out in our licence.

The Submitting Author accepts and understands that any supply made under these terms is made by BMJ to the Submitting Author unless you are acting as an employee on behalf of your employer or a postgraduate student of an affiliated institution which is paying any applicable article publishing charge ("APC") for Open Access articles. Where the Submitting Author wishes to make the Work available on an Open Access basis (and intends to pay the relevant APC), the terms of reuse of such Open Access shall be governed by a Creative Commons licence – details of these licences and which Creative Commons licence will apply to this Work are set out in our licence referred to above.

Other than as permitted in any relevant BMJ Author's Self Archiving Policies, I confirm this Work has not been accepted for publication elsewhere, is not being considered for publication elsewhere and does not duplicate material already published. I confirm all authors consent to publication of this Work and authorise the granting of this licence.

For peer review only

Sociodemographic differences in self-reported exposure to less healthy food and drink advertising: a cross-sectional analysis of UK panel data

Amy Yau[1], Jean Adams[2], Emma Boyland[3], Thomas Burgoine[2], Frank de Vocht[4,5], Matt Egan[6], Amelia A Lake[7,8], Karen Lock[9], Oliver Mytton[2], Mark Petticrew[6], Claire Thompson[10], Martin White[2], Steven Cummins[1]

1 Population Health Innovation Lab, Department of Public Health, Environments & Society, Faculty of Public Health & Policy, London School of Hygiene & Tropical Medicine, London, United Kingdom
2 Centre for Diet & Activity Research, MRC Epidemiology Unit, University of Cambridge, Cambridge, United Kingdom
3 Department of Psychology, Institute of Population Health, University of Liverpool, Liverpool, United Kingdom
4 Population Health Sciences, Bristol Medical School, University of Bristol, United Kingdom
5 National Institute for Health Research Applied Research Collaboration West (NIHR ARC West), Bristol, United Kingdom
6 Department of Public Health, Environments & Society, Faculty of Public Health & Policy, London School of Hygiene & Tropical Medicine, London, United Kingdom
7 Centre for Public Health Research, School of Health and Life Sciences, Teesside University, Middlesbrough, United Kingdom
8 Fuse, The Centre for Translational Research in Public Health, Newcastle upon Tyne, United Kingdom
9 Department of Health Services Research & Policy, Faculty of Public Health & Policy, London School of Hygiene & Tropical Medicine, London, United Kingdom
10 Centre for Research in Public Health and Community Care, School of Health and Social Work, University of Hertfordshire, Hatfield, United Kingdom

Corresponding author: Amy Yau (amy.yau@lshtm.ac.uk)

Word count: 3992

For peer review only - http://bmjopen.bmj.com/site/about/guidelines.xhtml

ABSTRACT

Objectives To explore sociodemographic differences in exposure to advertising for foods and drinks high in fat, salt and sugar (HFSS) and whether exposure is associated with body mass index (BMI).

Design Cross-sectional survey

Setting United Kingdom

Participants 1552 adults recruited to the Kantar Fast Moving Consumer Goods panel for London and the North of England

Outcome measures Self-reported advertising exposure stratified by product/service advertised (processed HFSS foods; sugary drinks; sugary cereals; sweet snacks; fast food or digital food delivery services) and advertising setting (traditional; digital; recreational; functional or transport); BMI and sociodemographic characteristics.

Results Overall, 84.7% of participants reported exposure to HFSS advertising in the past 7 days. Participants in the middle (vs high) socioeconomic group had higher odds of overall self-reported exposure (OR 1.48; 95% CI 1.06-2.07). Participants in the low (vs high) socioeconomic group had higher odds of reporting exposure to advertising for three of five product categories (ORs ranging from 1.36 to 1.67), advertising for digital food delivery services (OR 1.47; 95% CI 1.05-2.05), traditional advertising (OR 1.44; 95% CI 1.00-2.08) and digital advertising (OR 1.50; 95% CI 1.06-2.14). Younger adults (18-34 years vs ≥65 years)

For peer review only

had higher odds of reporting exposure to advertising for digital food delivery services (OR 2.08; 95% CI 1.20-3.59), digital advertising (OR 3.93; 95% CI 2.18-7.08) and advertising across transport networks (OR 1.96, 95% CI 1.11-3.48). Exposure to advertising for digital food delivery services (OR 1.40; 95% CI 1.05-1.88), digital advertising (OR 1.80; 95% CI 1.33-2.44) and advertising in recreational environments (OR 1.46; 95% CI 1.02-2.09) were associated with increased odds of obesity.

Conclusions Exposure to less healthy product advertising was prevalent, with adults in lower socioeconomic groups and younger adults more likely to report exposure. Broader restrictions may be needed to reduce sociodemographic differences in exposure to less healthy product advertising.

For peer review only

ARTICLE SUMMARY

Strengths and limitations of this study

- This study investigates exposure to advertising for a range of less healthy products in various settings, including some that have been less studied (e.g. advertising for digital food delivery services and advertising across transport networks)
- Self-reported exposure may not be an accurate reflection of actual exposure to advertising due to poor recall or social desirability bias
- The cross-sectional design of this study limits our interpretation of the findings as reverse causality may explain the associations we observed
- Participants were from two regions (London and the North of England), so may not be representative of the United Kingdom as a whole

For peer review only

For peer review only - http://bmjopen.bmj.com/site/about/guidelines.xhtml

INTRODUCTION

In recent years there has been an increased focus on the potential contribution of marketing and advertising of less healthy foods and drinks, such as those higher in fat, salt and sugar (HFSS), to population diet and diet-related disease.[1,2] Over £300 million was spent on advertising of less healthy foods and drinks in the UK in 2017.[3,4] This spend substantially outweighs that for healthier products. For example, £12 million was spent on advertising vegetables compared to almost £87 million on soft drinks in the UK in 2015.[4] Advertising and marketing of less healthy foods and drinks to children is widespread and is associated with children's preferences, requests for purchases, and consumption.[5–8] In adults, evidence is mixed, but some studies have found that exposure to less healthy food and drink advertising influences the purchasing and consumption of less healthy products and normalises the consumption of less healthy foods and drinks. [9–14] Advertising exposure may therefore be a plausible influence on obesity and diet-related non-communicable diseases.[15,16] A systematic review found that children from ethnic minority and socioeconomically disadvantaged backgrounds were disproportionately exposed to advertising for less healthy foods.[17] In adults, exposure to outdoor advertising and television advertising of less healthy foods are correlated with socioeconomic position in the UK.[18,19] These findings suggest that differential exposure to less healthy advertising may be contributing to the higher burden of obesity and diet-related non-communicable diseases among lower socioeconomic groups.[20–22]

In response, the regulation of food and drink marketing and advertising has been increasingly promoted as a policy lever for obesity prevention by local and national governments and the World Health Organisation.[8,23,24] In the UK, policies have focused

For peer review only - http://bmjopen.bmj.com/site/about/guidelines.xhtml

on highly-processed HFSS products as determined by the Nutrient Profile Model.[23,25] However, restrictions on other advertising may also be needed. The use and advertising of digital food delivery services have increased dramatically in recent years.[26] These services often deliver takeaway fast food or restaurant meals, which tend to be less healthy than home-prepared food.[27–29] Therefore, regulating the promotion of digital food delivery services may also be a potential policy lever to reduce consumption of less healthy products.

Obesity and diet quality are socially patterned, with greater prevalence of obesity and poorer diet quality associated with lower socioeconomic position.[21,30] Socioeconomic inequalities in exposure to advertising of less healthy foods and drinks may be a mechanism for the generation of socioeconomic inequalities in diet and obesity. While there is some evidence that levels of exposure to outdoor advertising and television advertising for HFSS products differ by socioeconomic position, the evidence base for sociodemographic correlates of advertising exposure in the UK remains limited.[18,19] Very little research has been conducted to explore differences in exposure to a wider range of advertising settings, such as digital advertising and advertising across transport networks.[31] Exposure to advertising for digital food delivery services is also poorly understood. Further, few studies have examined sociodemographic differences in advertising exposure beyond socioeconomic position. Differences in advertising exposure by other characteristics are likely. For example, by age due to marketing targeted at younger adults.[32] In addition, there has been limited work that directly explores associations between exposure to less healthy food and drink advertising and obesity. In this paper we address these gaps by using survey data from the UK (London and the North of England) to explore sociodemographic

For peer review only

For peer review only - http://bmjopen.bmj.com/site/about/guidelines.xhtml

differences in: (i) overall exposure to less healthy food and drink advertising, (ii) advertising exposure stratified by product category, (iii) exposure to advertising for digital food delivery services and (iv) advertising exposure stratified by advertising setting. We then examine the association between advertising exposure and body mass index (BMI).

METHODS

Data

We used baseline cross-sectional data from a study evaluating the impact of restricting advertising of HFSS foods and drinks across the Transport for London network.[33,34] Data were from households recruited to the UK Kantar Fast Moving Consumer Goods (FMCG) panel for London and the North of England (*n*=1552 households). Our sample size was based on the maximum number of available households within the panel for the two regions. The main food shopper from each recruited household was asked to complete an online survey (between 10th and 18th February 2019) on recent exposure to advertising of less healthy foods and drinks. The response rate was 71%. Households were recruited to the consumer panel through stratified random sampling and were representative of the regions from which they were sampled on the basis of household size, number of children, socioeconomic position and age of main shopper.[35,36] Panel households are recruited by Kantar through post and email.[36]

Ethical approval for this study was granted by the London School of Hygiene & Tropical Medicine Research Ethics Committee (ref no: 16297 /RR/11721).

Sociodemographic characteristics

For peer review only - http://bmjopen.bmj.com/site/about/guidelines.xhtml

Based on self-reported survey data, participants were categorised by sociodemographic characteristics: sex (male and female), age group (18-34 years, 35-44 years, 45-54 years, 55-64 years and ≥65 years), children <16 years in the household (yes or no), socioeconomic position and working status. For socioeconomic position, participants were classified according to the National Readership Survey (NRS) occupational social grade classification (A, B, C1, C2, D, E).[37] We categorised NRS social grade into three socioeconomic groups: high (AB), middle (C1C2) and low (DE). For working status, we categorised participants into six categories: full-time employee, part-time employee, self-employed, retired, not looking for work or unable to work (looking after home or family, long term sick or disabled, away from work due to illness, maternity leave, holiday or unemployed and not looking for work) or other (government sponsored training scheme, other paid work, student, actively looking for paid work or other).

Region

From household postcode data, participants were categorised as living in either London (Greater London) or the North of England (North West, North East or Yorkshire and the Humber).

Measuring exposure to advertising

We assessed exposure to advertising for five of the most commonly advertised food and drink product categories of current policy concern: processed HFSS foods, sugary drinks, sugary cereals, sweet snacks and fast food.[38] Definitions of the product categories used in the survey were adapted from the International Food Policy Study and are available in **Supplementary Table S1**.[39] In addition, exposure to advertising for digital food delivery

For peer review only

For peer review only - http://bmjopen.bmj.com/site/about/guidelines.xhtml

services was assessed with a question on exposure to advertising for food delivery apps, with the market-leading services (Uber Eats, Deliveroo, Just Eat and FoodHub) listed as examples.

Survey questions were structured as follows: *"In the last 7 days, have you seen or heard advertisements for* [category] *in the following places?"*. Within each question, 19 places where advertisements may have been seen or heard were specified. Using methods adapted from Forde et al,[12] we re-categorised the 19 places into five advertising settings prior to analysis: traditional advertising, digital advertising, advertising in recreational environments, advertising in functional environments and advertising across transport networks (**Box 1**). Participants were also able to report other places using free text. We allocated these text responses into the five advertising settings, treating the categories as mutually exclusive. Where there was ambiguity in categorisation, advertising setting was categorised based on *where* the advertisement was seen or heard rather than what medium was used. For example, a digital advertisement *at a bus stop* was categorised as transport.

For peer review only - http://bmjopen.bmj.com/site/about/guidelines.xhtml

121 **Box 1.** Categorisation of advertising setting

Advertising setting	Description	Included survey responses
Traditional	Physical, non-digital text and radio media and direct marketing	Television, radio, text message, newspaper/magazine, email and leaflet
Digital	Advertising seen or heard through digital platforms and social media	Online/internet, mobile app, video game and social media
Recreational	Advertising placed in environments that people interact with for enjoyment and leisure purposes	Film/cinema, leisure centre/gym/community centre, sports event/concert/community event, giveaway/sample/special offer and pub
Functional	Advertising placed in environments that people visit for a specific purposes and to complete a specific task (e.g. school, work and shops)	Billboard/outdoor signs, telephone boxes, school/college/university, signs or displays in supermarket/convenience stores/restaurants, delivery drivers, doctor's surgery, shopping centre and motorway services
Transport	Advertising placed in environments related to transport	Outside/inside buses, outside/inside tube, tram or train, outside/inside of tube or train station, bus stop, taxi and back of bus ticket

122

123 We coded advertising exposure into two categories: exposed or not. For overall exposure,
124 participants who reported seeing or hearing advertisements for any of the six
125 product/service categories (processed HFSS foods; sugary drinks; sugary cereals; sweet
126 snacks; fast food and digital food delivery services) were classified as exposed.

127

128 **BMI and weight status**

129 BMI (weight/height^2) was calculated using self-reported height and weight data. BMI was
130 available for 81.7% of participants (*n*=1268), who were then classified into four categories:
131 underweight (BMI<18.5), normal (BMI 18.5-25), overweight (BMI 25-30) and obese (BMI
132 ≥30). A fifth category contained participants with missing BMI data (*n*=284).

133

134 **Statistical methods**

For peer review only

For peer review only - http://bmjopen.bmj.com/site/about/guidelines.xhtml

We calculated the number (%) of participants reporting exposure to less healthy food and drink advertising (including digital food delivery services) in the past 7 days, overall and stratified by product category and advertising setting. Using logistic regression models we estimated odds ratios, with 95% confidence intervals (CIs), for the association between sociodemographic characteristics and advertising exposure. As advertising exposure may be influenced by social and environmental factors, independently or in combination, we tested for interactions between sociodemographic characteristics and region (London or the North of England). Separate models were used to look at overall advertising exposure, advertising exposure stratified by product category, exposure to advertising for digital food delivery services and exposure stratified by advertising setting. We also used logistic regression models to investigate the association between self-reported advertising exposure and odds of living with overweight or obesity. All regression models were mutually adjusted for sex, age group, socioeconomic position, children in the household, working status and region. All analyses were conducted in Stata IC version 16 and completed in September 2020.

Patient and public involvement

Patients and the public were not involved in the design, or conduct, or reporting, or dissemination plans of our research.

RESULTS

Overall, 1552 participants were included in this study (**Table 1**) with 45.4% of participants living in London and 54.6% in the North of England. The majority of participants were female (71.6%), in the middle socioeconomic group (C1C2) (59.7%), had no children <16 years in the household (71.5%) and were in work (64.1%).

For peer review only

Self-reported exposure to advertising

Overall, 84.7% of participants reported seeing or hearing advertising for less healthy foods and drinks and/or digital food delivery services in the past 7 days (see **Supplementary Table S2**). The proportion of participants reporting exposure to advertising differed according to product category: 68.2% for processed HFSS foods, 52.4% for sugary drinks, 42.1% for sugary cereals, 55.0% for sweet snacks, 71.3% for fast food and 54.9% for digital food delivery services. Reported exposure also varied according to advertising setting: 74.0% of participants reported seeing or hearing traditional advertising, 38.7% digital advertising, 18.8% advertising in recreational environments, 51.5% advertising in functional environments and 36.4% advertising across transport networks (see **Supplementary Table S3**). For most product categories, reported exposure was most common through traditional advertising, followed by advertising in functional environments, digital advertising, advertising across transport networks, and lastly advertising in recreational environments. However, for digital food delivery services, digital was the second highest reported setting after traditional. Self-reported exposure to advertising was higher in London than the North of England for sugary drinks (55.3% vs 49.9%, P=0.035), digital food delivery services (59.0% vs 51.5%, P=0.003) and advertising across transport networks (45.5% vs 28.8%, P<0.001) (see **Supplementary Table S4)**. After adjustments, participants living in London had higher odds of self-reported exposure for sugary drinks (OR 1.27; 95% CI 1.03-1.56), digital food delivery services (OR 1.39; 95% 1.13-1.71) and advertising across transport networks (OR 2.05; 95% CI 1.65-2.54) compared to those living in the North of England (**Table 2-3**).

For peer review only

For peer review only - http://bmjopen.bmj.com/site/about/guidelines.xhtml

Sociodemographic differences in self-reported exposure to advertising for less healthy foods and drinks

The adjusted odds of self-reported exposure to advertising overall were higher in the middle socioeconomic group (C1C2) compared to the high socioeconomic group (AB) (OR 1.48; 95% CI 1.06-2.07) (**Table 2**). When stratified by product category, sociodemographic differences in self-reported exposure to advertising were found in three of the five categories studied (processed HFSS foods, sugary cereals and sweet snacks). Socioeconomic differences were observed for these three product categories, with lower socioeconomic position associated with higher adjusted odds of self-reported exposure (ORs ranging from 1.41 to 1.67 for low compared to high). There was indication of a socioeconomic gradient for all three product categories, although the CIs for the middle and low socioeconomic groups overlapped. Gender differences were also observed for processed HFSS foods, with higher adjusted odds for women compared to men (OR 1.44; 95% CI 1.13-1.84). For sweet snacks, participants who were full-time employees had marginally higher adjusted odds of reporting advertising exposure compared to participants who were not looking for work or unable to work (OR 1.40; 95% CI 1.00-1.97). There were no observed associations between sociodemographic characteristics and self-reported exposure to advertising for fast food or sugary drinks. There were some interactions between sociodemographic characteristics and region on their influence on self-report advertising exposure (see **Supplementary Table S5**). Results stratified by region are presented in **Supplementary Tables S6 and S7** where significant.

Sociodemographic differences in exposure to advertising for digital food delivery services

Higher adjusted odds of reporting exposure to advertising for digital food delivery services were observed in the lower socioeconomic groups compared to the highest: low (OR 1.47;

For peer review only

95% CI 1.05-2.05) and middle (OR 1.39; 95% CI 1.08-1.80). The adjusted odds were also higher among participants aged 18-34 years (OR 2.08; 95% CI 1.20-3.59), 35-44 years (OR 1.93; 95% CI 1.15-3.36) and 55-64 years (OR 1.53; 95% CI 1.00-2.35) compared to those aged ≥65 years.

Sociodemographic differences in exposure to advertising by advertising setting

Sociodemographic differences in self-reported exposure were found for traditional advertising, digital advertising and advertising across transport networks (**Table 3**). Adjusted odds of self-reported exposure were higher in the lower socioeconomic groups compared to the high socioeconomic group for traditional advertising: low (OR 1.44; 95% CI 1.00-2.08) and middle (OR 1.52; 95% CI 1.15-2.00). Adjusted odds of self-reported exposure to digital advertising were higher in the low compared to high socioeconomic group (OR 1.50; 95% CI 1.06-2.14), and younger age groups compared to ≥65 years: 18-34 years (OR 3.93, 95% CI 2.18-2.07) and 35-44 years (OR 3.06; 95% CI 1.74-5.40). The adjusted odds of reporting exposure to advertising across transport networks were higher among participants aged 18-34 years compared to ≥65 years (OR 1.96; 95% CI 1.11-3.48), and participants who were full-time employees compared to those not looking for work or unable to work (OR 1.50; 95% CI 1.04-2.17).

Association between advertising exposure and BMI

Overall self-reported exposure to less healthy food and drink advertising was not associated with BMI category (**Table 4**). However, higher adjusted odds of living with obesity were observed among participants who reported exposure to advertising for digital food delivery

For peer review only - http://bmjopen.bmj.com/site/about/guidelines.xhtml

services (OR 1.40; 95% CI 1.05-1.88), digital advertising (OR 1.80; 95% CI 1.33-2.44) and advertising in recreational environments (OR 1.46; 95% CI 1.02-2.09).

DISCUSSION

Summary of findings

This is one of the few studies to investigate sociodemographic correlates of exposure to less healthy food and drink advertising in UK adults. Overall, exposure was high, with 84.7% of participants reporting seeing or hearing advertising for less healthy foods and drinks and/or digital food delivery services in the past 7 days. Lower socioeconomic position was associated with increased odds of advertising exposure. Overall, the middle (C1C2) socioeconomic group had higher odds of exposure compared to the most affluent group (AB). For three of the five food and drink product categories (processed HFSS foods, sugary cereals and sweet snacks) and digital food delivery services, increased odds of self-reported exposure to advertising were observed for lower socioeconomic groups (DE and C1C2 compared to AB). When stratified by advertising setting, higher odds of reporting exposure among lower socioeconomic groups were observed for traditional (C1C2 and DE) and digital advertising (DE only). Younger participants had higher odds of self-reported exposure to advertising for digital food delivery services compared to participants aged ≥65 years, except those aged 45-54 years. Younger participants were also more likely to report exposure to digital advertising (18-34 and 35-44 age groups) and advertising across transport networks (18-34 age group). Participants who were full-time employees had higher self-reported odds of exposure to advertising for sweet snacks and advertising across transport networks. Women had higher odds of reporting exposure to advertising for processed HFSS foods than men. Exposure to advertising for digital food delivery services,

For peer review only - http://bmjopen.bmj.com/site/about/guidelines.xhtml

digital advertising and advertising in recreational environments were associated with obesity.

Strengths & limitations

Whilst most of the existing research on less healthy food and drink advertising focuses on children, this study adds to the evidence for sociodemographic differences in advertising exposure in adults. There are some strengths and limitations of this study that should be noted. There is no standard method for measuring advertising exposure. The questions used in this study were informed by those used in the International Food Policy Study.[12] The survey questions asked about the products advertised and the advertising setting, giving us the opportunity to explore what was advertised and where. This provided a broader perspective relative to studies focusing on one form of advertising.[18,19] The inclusion of digital food delivery services in the survey also provided insight into a fast-growing and understudied channel for purchasing less healthy foods and drinks.

The cross-sectional nature of this study and short recall period may mean that exposure as measured is not representative of typical exposure, for example due to the seasonality of advertising.[41] Self-reported data may also be subject to misreporting. Participants may not have recalled all advertising they had seen or heard, resulting in under-reporting. We also did not account for the intensity of exposure, as we only categorised participants as exposed or not, and we did not measure how many advertisements participants saw or heard. The cross-sectional nature of this study also limits our ability to establish causality and reverse causation could explain some of the associations observed.

For peer review only - http://bmjopen.bmj.com/site/about/guidelines.xhtml

Generalisability

Our study sample was likely representative of populations in London and the North of England, as Kantar assesses representativeness of their panels every four weeks.[36] However, our study sample was not representative of the UK as a whole. Nonetheless, our study sample appears similar to national samples and the general population in terms of distribution of most sociodemographic characteristics. Most participants (71.6%) were female, which is likely due to more women being the main food shopper within a household than men. A similar proportion of main food providers were women (73.3%) in latest wave (2014/15 to 2016/17) of the National Diet and Nutrition Survey (NDNS), which aims to be representative of the UK population.[44] A similar proportion of participants also reported not having children in the household in our sample (71.5%) as in the NDNS (69.8%), but this is higher than in the general population (58.0%).[45] Having children may impact on advertising exposure (e.g. through different advertisements shown around children's television programmes) or recall of advertising exposure (e.g. through 'pester power').[46,47] A greater proportion of participants in our sample reported being in work (62.2%) than in the NDNS (56.2%). Discounting those with missing BMIs, the proportion of participants in each BMI category in this study was similar to that found in the Health Survey for England 2018.[21] However, the number of participants in the underweight category was too small for any statistically meaningful interpretations to be drawn for this group in our study.

Interpretation

Socioeconomic differences in self-reported advertising exposure were observed across various product categories and advertising settings, with more disadvantaged groups more

For peer review only - http://bmjopen.bmj.com/site/about/guidelines.xhtml

likely to report exposure than the least disadvantaged group. These findings are consistent with previous studies that documented socioeconomic differences in exposure to less healthy food and drink advertising in the UK using more objective measures of exposure.[18,19] The alignment between objective and self-reported measures suggests that self-reported measures may be appropriate proxies for advertising exposure. Further, our findings are consistent with those in the wider international literature.[17,48,49]

There are various possible explanations for the associations between sociodemographic characteristics and self-reported advertising exposure observed in this study (**Figure 1**). Low socioeconomic position, younger age, full-time working and being female may be driving higher exposure to less healthy advertising. For example, for socioeconomic position, this could be through a greater concentration of advertising for less healthy products in more disadvantaged areas. Studies in the US have found that advertising for HFSS foods and drinks was more prevalent in more deprived neighbourhoods,[19] and that the types of products advertised also differ depending on neighbourhood demographics.[50] We did not capture the urbanicity of participants' locations in this study, which may also influence the density and types of outdoor advertising present in the local neighbourhood.[50] Another potential influence on actual advertising exposure is differential use of public transport across population subgroups. Participants who were full-time employees were more likely to report exposure to advertising across transport networks. This may be because these participants were more likely to use public transport to get to work. Similarly, younger adults may have been more exposed to advertising across transport networks due to more frequent public transport use.[51,52] Differences in advertising exposure could also be

For peer review only

associated with known sociodemographic differences in screen time and use of certain media, such as radio and social media.[53,54]

Alternatively, the observed differences may be due to differences in recall rather than actual exposure. The content of advertisements may be designed to appeal to certain population groups resulting in different cognitive responses to advertisements. Identity salience (defined as heightened sensitivity to identity-relevant stimuli) may make advertisements more impactful to certain groups (i.e. advertisements that feel relevant may be more impactful) and therefore more memorable.[55] Some groups may also be more receptive to advertising due to higher cognitive load. One experiment showed greater effect of advertising on food choices when cognitive load was higher.[11] The authors argued that the effect of advertising on food choices could therefore be exacerbated in low socioeconomic groups because of the greater cognitive load of daily pressures. Receptiveness to advertising may also decrease with age.[32] Equally, sociodemographic differences in reporting may reflect differential social desirability bias across population groups.

Differences in reported exposure to advertising across transport networks between London and the North of England may be partly explained by differences in the extent of public transport infrastructure. London has the most extensive transport network in the UK and accounts for a large amount of advertising space.[33] Participants living in London also reported greater exposure to advertising for digital food delivery services. This is may be due to the greater penetration of digital food delivery services in London while these services are continuing to expand.[56]

For peer review only - http://bmjopen.bmj.com/site/about/guidelines.xhtml

Self-reported exposure to advertising for digital food delivery services, digital advertising and advertising in recreational environments were independently associated with higher odds of obesity. Whilst it is implausible that advertising exposure over seven days directly caused obesity, advertising exposure may be stable over time and suggestive of a long-term effect of advertising on the consumption of less healthy products. Reverse causality, whereby people living with obesity are more likely to see or hear, recall or report advertising of less healthy products, may also explain the associations observed.

A large majority of participants reported seeing or hearing some form of advertising for less healthy foods and drinks and/or digital food delivery services, indicating high prevalence of exposure across the UK adult population. Companies often use a variety of advertising strategies to reach a wide audience and reinforce advertising through consistent messaging across settings.[59] This suggests that restrictions may need to be broadened beyond those proposed in the government's plans to tackle obesity.[60] Digital food delivery services were disproportionately advertised through digital channels, meaning that exposure to such advertising can be readily accessed from any location. Exposure to fast food advertising was the most commonly reported of the product categories studied and no sociodemographic variation was observed, which may indicate the pervasiveness of fast food advertising in particular.

Going forward, research evaluating the impacts of policies to restrict advertising of less healthy foods and drinks should be a priority in order to better understand the utility of such policies. Research comparing methods of measuring advertising exposure may also be

For peer review only - http://bmjopen.bmj.com/site/about/guidelines.xhtml

beneficial and the development of a standardised method could aid monitoring and evaluation across different settings. Longitudinal studies of advertising exposure and consumption of less healthy foods and drinks would help to determine whether the relationship is causal. If so, interventions should be designed to harness the potential for advertising restrictions to reduce advertising exposure in the groups that are most exposed.

Conclusions

Exposure to advertising of less healthy foods and drinks was highly prevalent, with adults in lower socioeconomic groups and younger adults more likely to report exposure. Therefore, broader advertising restrictions may be needed to reduce sociodemographic differences in exposure to less healthy product advertising.

List of abbreviations

BMI	Body Mass Index
CI(s)	Confidence Interval(s)
FMCG	Fast Moving Consumer Goods
HFSS	High fat, salt and sugar
NDNS	National Diet and Nutrition Survey
NRS	National Readership Survey
UK	United Kingdom

For peer review only

DECLARATIONS

Ethics approval and consent to participate

Ethical approval for this study was granted by the London School of Hygiene & Tropical Medicine Research Ethics Committee (Application No. 16297 /RR/11721). Written informed consent was obtained from all participants.

Consent for publication

Not applicable.

Availability of data and materials

Data were collected via an online survey linked to social and demographic data from Kantar WorldPanel Plus. The terms of our data agreement with Kantar mean that we cannot share linked social and demographic data, however access to the online survey data for the purposes of replication only may be possible. Please contact the study Principal Investigator, Professor Steven Cummins (steven.cummins@lshtm.ac.uk).

Competing interests

The authors declare that they have no competing interests.

Funding

The National Institute for Health Research (NIHR) School for Public Health Research is a partnership between the Universities of Sheffield; Bristol; Cambridge; Imperial; and University College London; The London School for Hygiene and Tropical Medicine (LSHTM); LiLaC – a collaboration between the Universities of Liverpool and Lancaster; and Fuse - The

For peer review only

Centre for Translational Research in Public Health, a collaboration between Newcastle, Durham, Northumbria, Sunderland and Teesside Universities. This study is funded by the NIHR School for Public Health Research (SPHR) (Grant Reference Number PD-SPH-2015). SC is also funded by Health Data Research UK (HDR-UK). HDR-UK is an initiative funded by the UK Research and Innovation, Department of Health and Social Care (England) and the devolved administrations, and leading medical research charities. JA, MW and TB are supported by the MRC Epidemiology Unit, University of Cambridge [grant number MC/UU/12015/6] and Centre for Diet and Activity Research (CEDAR), a UK Clinical Research Collaboration (UKCRC) Public Health Research Centre of Excellence. Funding for CEDAR from the British Heart Foundation, Cancer Research UK, Economic and Social Research Council, Medical Research Council, the National Institute for Health Research [grant numbers ES/G007462/1 and MR/K023187/1], and the Wellcome Trust [grant number 087636/Z/08/Z], under the auspices of the UK Clinical Research Collaboration, is gratefully acknowledged. CT is funded by the NIHR Applied Research Collaboration East of England. AAL is a member of Fuse, the Centre for Translational Research in Public Health (www.fuse.ac.uk). Fuse is a Public Health Research Centre of Excellence funded by the five North East Universities of Durham, Newcastle, Northumbria, Sunderland and Teesside. FdV is partly funded by NIHR Applied Research Collaboration West (NIHR ARC West) at University Hospitals Bristol NHS Foundation Trust. The views expressed are those of the authors and do not necessarily represent those of any of the above named funders. The funders had no role in the design of the study, or collection, analysis and interpretation of the data, or in the decision to publish, or in writing the manuscript.

Authors' contributions

For peer review only

All of the authors contributed to the development of the research questions and the design of the study. AY led on the analysis with support from SC. AY led on the writing of the manuscript with support from SC. All authors critically reviewed and edited the manuscript. All authors read and approved the final manuscript.

Acknowledgements

Not applicable.

For peer review only

REFERENCES

1. World Health Organization. Marketing of foods high in fat, salt and sugar to children: update 2012–2013 [Internet]. 2013. Available from: http://www.euro.who.int/pubrequest

2. HM Government. Introducing further advertising restrictions on TV and online for products high in fat, sugar and salt (HFSS) [Internet]. London; 2019. Available from: https://assets.publishing.service.gov.uk/government/uploads/system/uploads/attachment_data/file/807378/hfss-advertising-consultation-10-april-2019.pdf

3. The Food Foundation. The Broken Plate. London; 2019.

4. The Food Foundation. Veg Facts A briefing by the Food Foundation [Internet]. London; 2016. Available from: https://foodfoundation.org.uk/wp-content/uploads/2016/11/FF-Veg-Doc-V5.pdf

5. Boyland EJ, Nolan S, Kelly B, Tudur-Smith C, Jones A, Halford JCG, et al. Advertising as a cue to consume: A systematic review and meta-analysis of the effects of acute exposure to unhealthy food and nonalcoholic beverage advertising on intake in children and adults. Am J Clin Nutr. American Society for Nutrition; 2016;103:519–33.

6. Sadeghirad B, Duhaney T, Motaghipisheh S, Campbell NRC, Johnston BC. Influence of unhealthy food and beverage marketing on children's dietary intake and preference: a systematic review and meta-analysis of randomized trials. Obes Rev. 2016;17:945–59.

7. Aschemann-Witzel J, Perez-Cueto FJ, Niedzwiedzka B, Verbeke W, Bech-Larsen T. Lessons for public health campaigns from analysing commercial food marketing success factors: A case study. BMC Public Health [Internet]. 2012;12. Available from: https://www.scopus.com/inward/record.uri?eid=2-s2.0-84857135656&doi=10.1186%2F1471-2458-12-139&partnerID=40&md5=e198945e820f92d04f46948dfc46c8dc

8. World Health Organisation. Reducing the impact of marketing of foods and non-alcoholic beverages on children [Internet]. 2019 [cited 2020 Jul 28]. Available from: https://www.who.int/elena/titles/food_marketing_children/en/

9. Harris JL, Bargh JA, Brownell KD. Priming Effects of Television Food Advertising on Eating Behavior. Heal Psychol. 2009;28:404–13.

10. Koordeman R, Anschutz DJ, van Baaren RB, Engels RCME. Exposure to soda commercials affects sugar-sweetened soda consumption in young women. An observational

For peer review only

experimental study. Appetite [Internet]. Elsevier Ltd; 2010;54:619–22. Available from: http://dx.doi.org/10.1016/j.appet.2010.03.008

11. Zimmerman FJ, Shimoga S V. The effects of food advertising and cognitive load on food choices. BMC Public Health. 2014;14:342.

12. Forde H, White M, Levy L, Greaves F, Hammond D, Vanderlee L, et al. The relationship between self-reported exposure to sugar-sweetened beverage promotions and intake: Cross-sectional analysis of the 2017 international food policy study. Nutrients. 2019;11:3047.

13. Critchlow N, Bauld L, Thomas C, Hooper L, Vohra J. Awareness of marketing for high fat, salt, or sugar (HFSS) foods, and the association with higher weekly consumption among adolescents: A rejoinder to the UK Government's consultations on marketing regulation. Public Health Nutr. 2020;

14. Cairns G. A critical review of evidence on the sociocultural impacts of food marketing and policy implications. Appetite [Internet]. Elsevier; 2019;136:193–207. Available from: https://doi.org/10.1016/j.appet.2019.02.002

15. OECD. Obesity Update 2017 [Internet]. Paris; 2017. Available from: www.oecd.org/health/obesity-update.htm

16. Baker C. House of Commons Library | Obesity Statistics. Obes. Stat. (Briefing Pap. Number 3336). London: House of Commons; 2019.

17. Backholer K, Gupta A, Zorbas C, Bennett R, Huse O, Chung A, et al. Differential exposure to, and potential impact of, unhealthy advertising to children by socio-economic and ethnic groups: A systematic review of the evidence. Obes Rev. 2020;

18. Adams J, Tyrrell R, Adamson AJ, White M. Socio-economic differences in exposure to television food advertisements in the UK: a cross-sectional study of advertisements broadcast in one television region. Public Health Nutr [Internet]. 2011 [cited 2019 Aug 22];15:487–94. Available from: http://www.nrs.co.uk/lifestyle.htm

19. Adams J, Ganiti E, White M. Socio-economic differences in outdoor food advertising in a city in Northern England. Public Health Nutr [Internet]. Cambridge University Press; 2011 [cited 2019 Aug 23];14:945–50. Available from: https://www.cambridge.org/core/product/identifier/S1368980010003332/type/journal_article

20. Steel N, Hardcastle AC, Bachmann MO, Richards SH, Mounce LTA, Clark A, et al.

For peer review only

For peer review only - http://bmjopen.bmj.com/site/about/guidelines.xhtml

Economic inequalities in burden of illness, diagnosis and treatment of five long-term conditions in England: Panel study. BMJ Open. 2014;4:e005530.

21. NHS Digital. Health Survey for England 2018: Data tables. 2019.

22. Lewer D, Jayatunga W, Aldridge RW, Edge C, Marmot M, Story A, et al. Premature mortality attributable to socioeconomic inequality in England between 2003 and 2018: an observational study. Lancet Public Heal [Internet]. 2019 [cited 2019 Dec 8];5:PE33-E41. Available from: https://linkinghub.elsevier.com/retrieve/pii/S2468266719302191

23. Chambers SA, Freeman R, Anderson AS, MacGillivray S. Reducing the volume, exposure and negative impacts of advertising for foods high in fat, sugar and salt to children: A systematic review of the evidence from statutory and self-regulatory actions and educational measures. Prev Med (Baltim) [Internet]. Elsevier Inc.; 2015;75:32–43. Available from: http://dx.doi.org/10.1016/j.ypmed.2015.02.011

24. Kovic Y, Noel JK, Ungemack JA, Burleson JA. The impact of junk food marketing regulations on food sales: an ecological study. Obes Rev. 2018;19:761–9.

25. World Health Organization. Evaluating implementation of the WHO set of recommendations on the marketing of foods and non-alcoholic beverages to children: Progress, challenges and guidance for next steps in the WHO European Region [Internet]. Copenhagen; 2018. Available from: http://www.euro.who.int/__data/assets/pdf_file/0003/384015/food-marketing-kids-eng.pdf

26. Pollack CC, Kim J, Emond JA, Brand J, Gilbert-Diamond D, Masterson TD. Prevalence and strategies of energy drink, soda, processed snack, candy and restaurant product marketing on the online streaming platform Twitch. Public Health Nutr. 2020;23:2793–803.

27. Jaworowska A, Blackham T, Stevenson L, Davies IG. Determination of salt content in hot takeaway meals in the United Kingdom. Appetite. 2012;59:517–22.

28. Jaworowska A, Blackham T, Long R, Taylor C, Ashton M, Stevenson L, et al. Nutritional composition of takeaway food in the UK. Nutr Food Sci. 2014;44:414–30.

29. Robinson E, Jones A, Whitelock V, Mead BR, Haynes A. (Over)eating out at major UK restaurant chains: Observational study of energy content of main meals. BMJ. 2018;363:k4982.

30. Yau A, Adams J, Monsivais P. Time trends in adherence to UK dietary recommendations and associated sociodemographic inequalities, 1986-2012: a repeated cross-sectional

For peer review only - http://bmjopen.bmj.com/site/about/guidelines.xhtml

analysis. Eur J Clin Nutr [Internet]. 2019 [cited 2019 May 21];73:997–1005. Available from: http://www.nature.com/articles/s41430-018-0347-z

31. Mills SDH, Tanner LM, Adams J. Systematic literature review of the effects of food and drink advertising on food and drink-related behaviour, attitudes and beliefs in adult populations. Obes Rev. 2013;14:303–14.

32. Freeman B, Kelly B, Vandevijvere S, Baur L. Young adults: Beloved by food and drink marketers and forgotten by public health? Health Promot Int. 2016;31:954–61.

33. Transport for London. Advertising Annual Report 2017/18. London; 2018.

34. Cummins S. Restricting advertising of high fat, salt and sugar foods and drinks on the Transport for London network: evaluation of a natural experiment [Internet]. 2019. Available from: http://www.isrctn.com/ISRCTN19928803

35. Berger N, Cummins S, Smith RD, Cornelsen L. Recent trends in energy and nutrient content of take-home food and beverage purchases in Great Britain: an analysis of 225 million food and beverage purchases over 6 years. BMJ Nutr Prev Heal. 2019;2:63–71.

36. Cornelsen L, Berger N, Cummins S, Smith RD. Socio-economic patterning of expenditures on 'out-of-home' food and non-alcoholic beverages by product and place of purchase in Britain. Soc Sci Med [Internet]. Elsevier; 2019;235:112361. Available from: https://doi.org/10.1016/j.socscimed.2019.112361

37. National Readership Survey. Social Grade [Internet]. 2018 [cited 2020 Sep 22]. Available from: http://www.nrs.co.uk/nrs-print/lifestyle-and-classification-data/social-grade/

38. Cairns G, Angus K, Hastings G, Caraher M. Systematic reviews of the evidence on the nature, extent and effects of food marketing to children. A retrospective summary. Appetite. 2013.

39. Hammond D. International Food Policy Study [Internet]. 2019. Available from: http://foodpolicystudy.com/about/

40. Transport for London. TfL Ad Policy : Approval Guidance Food and Non-Alcoholic Drink Advertising [Internet]. London; 2019. Available from: http://content.tfl.gov.uk/policy-guidance-food-and-drink-advertising.pdf

41. Adams J, Simpson E, White M. Variations in food and drink advertising in UK monthly women's magazines according to season, magazine type and socio-economic profile of readers: A descriptive study of publications over 12 months. BMC Public Health. 2011;11:368.

For peer review only - http://bmjopen.bmj.com/site/about/guidelines.xhtml

42. Harris JL, Brownell KD, Bargh JA. The Food Marketing Defense Model: Integrating Psychological Research to Protect Youth and Inform Public Policy. Soc Issues Policy Rev. 2009;3:211–71.

43. Boyland E, Tatlow-Golden M. Exposure, Power and Impact of Food Marketing on Children: Evidence Supports Strong Restrictions. Eur J Risk Regul. 2017;8:224–36.

44. NatCen Social Research. MRC Elsie Widdowson Laboratory. National Diet and Nutrition Survey Years 1-9, 2008/09-2016/17. [data collection]. 15th Edition. [data collection]. 15th Edition; 2019.

45. Office for National Statistics. Families and households in the UK: 2019. Southampton: ONS; 2019.

46. Huang CY, Reisch LA, Gwozdz W, Molnár D, Konstabel K, Michels N, et al. Pester power and its consequences: Do European children's food purchasing requests relate to diet and weight outcomes? Public Health Nutr. 2016;19:2393–403.

47. Whalen R, Harrold J, Child S, Halford J, Boyland E. The health halo trend in UK television food advertising viewed by children: The rise of implicit and explicit health messaging in the promotion of unhealthy foods. Int J Environ Res Public Health. 2018;15.

48. Kumanyika S, Grier S. Targeting interventions for ethnic minority and low-income populations. Futur Child. 2006;16:187–207.

49. Lucan SC, Maroko AR, Sanon OC, Schechter CB. Unhealthful Food-and-Beverage Advertising in Subway Stations: Targeted Marketing, Vulnerable Groups, Dietary Intake, and Poor Health. J Urban Heal. Journal of Urban Health; 2017;94:220–32.

50. Kelly B, Cretikos M, Rogers K, King L. The commercial food landscape: Outdoor food advertising around primary schools in Australia. Aust N Z J Public Health. 2008;32:522–8.

51. Campaign for Better Transport. Why getting transport right matters to young people [Internet]. London; 2013. Available from: http://www.bettertransport.org.uk/files/Young_People_and_Buses_FINAL_forweb_0.pdf

52. Transport for London. TfL Bus User Survey 2014. London; 2014.

53. Stamatakis E, Coombs N, Rowlands A, Shelton N, Hillsdon M. Objectively-assessed and self-reported sedentary time in relation to multiple socioeconomic status indicators among adults in England: A cross-sectional study. BMJ Open. 2014;4:1–10.

54. Ofcom. Adults' media use and attitudes report [Internet]. London; 2019. Available from: http://stakeholders.ofcom.org.uk/market-data-research/media-literacy/media-lit-

For peer review only - http://bmjopen.bmj.com/site/about/guidelines.xhtml

research/adults-2013/

55. Forehand MR, Deshpandé R, Reed A. Identity salience and the influence of differential activation of the social self-schema on advertising response. J Appl Psychol. 2002;87:1086–99.

56. Just Eat Group. 2018 Capital Markets Day. London; 2018.

57. Deloitte. Delivering growth: The impact of third-party platform ordering on restaurants. London; 2019.

58. Keeble M, Adams J, Sacks G, Vanderlee L, White CM, Hammond D, et al. Use of online food delivery services to order food prepared away-from-home and associated sociodemographic characteristics: A cross-sectional, multi-country analysis. Int J Environ Res Public Health. 2020;17:5190.

59. Montgomery KC, Chester J. Interactive Food and Beverage Marketing: Targeting Adolescents in the Digital Age. J Adolesc Heal [Internet]. Elsevier Ltd; 2009;45:S18–29. Available from: http://dx.doi.org/10.1016/j.jadohealth.2009.04.006

60. Department of Health and Social Care: Global Public Health Directorate: Obesity Food and Nutrition / 10800. Childhood obesity: a plan for action Chapter 2 [Internet]. London: HM Government; 2018 [cited 2018 Aug 2]. Available from: www.nationalarchives.gov.uk/doc/open-government-licence/

For peer review only

FIGURE CAPTIONS

Figure 1. Potential explanations for sociodemographic differences in exposure to advertising for less healthy foods and drinks

For peer review only

Table 1. Sociodemographic characteristics of study population (*n*=1552)

Sociodemographic characteristic		Total, N (%)	London, N (%)	North, N (%)	X^2 (P-Value)
Sex	Male	441 (28.4)	213 (30.2)	228 (26.9)	2.05 (0.152)
	Female	1,111 (71.6)	492 (69.8)	619 (73.1)	
Age group, years	18-34	188 (12.1)	65 (9.20)	123 (14.5)	18.73 (0.001)***
	35-44	299 (19.3)	160 (22.7)	139 (16.4)	
	45-54	411 (26.5)	196 (27.8)	215 (25.4)	
	55-64	335 (21.6)	145 (20.6)	190 (22.4)	
	≥65	319 (20.6)	139 (19.7)	180 (21.3)	
Socioeconomic position	AB	341 (22.0)	177 (25.1)	164 (19.4)	7.92 (0.019)*
	C1C2	926 (59.7)	409 (58.0)	517 (61.0)	
	DE	285 (18.4)	119 (16.9)	166 (19.6)	
Children in the household	No	1,110 (71.5)	507 (71.9)	603 (71.2)	0.10 (0.754)
	Yes	442 (28.5)	198 (28.1)	244 (28.8)	
Working status	Full-time	612 (39.4)	285 (40.4)	327 (36.8)	19.88 (0.001)***
	Part-time	223 (14.4)	91 (12.9)	132 (15.6)	
	Self-employed	131 (8.4)	78 (11.6)	53 (6.3)	
	Retired	342 (22.0)	145 (20.6)	197 (23.3)	
	Not looking/ unable to work	214 (13.8)	87 (12.3)	127 (15.0)	
	Other	30 (1.9)	19 (2.7)	11 (1.3)	
BMI	Underweight	31 (2.0)	21 (3.0)	10 (1.2)	11.91 (0.018)*
	Normal	479 (30.9)	236 (33.5)	243 (28.7)	
	Overweight	425 (27.4)	180 (25.5)	245 (28.9)	
	Obese	336 (21.7)	143 (20.3)	193 (22.8)	
	Missing	281 (18.1)	125 (17.7)	156 (18.4)	
Region	North	847 (54.6)	N/A	N/A	N/A
	London	705 (45.4)			

For peer review only

For peer review only - http://bmjopen.bmj.com/site/about/guidelines.xhtml

Table 2. Sociodemographic correlates of advertising exposure stratified by product/service advertised (*n*=1552)

Sociodemographic characteristic		Any	Processed HFSS foods	Sugary drinks	Sugary cereals	Sweet snacks	Fast food	Digital food delivery services
Sex	Male	REF	REF	REF	REF	REF	REF	REF
	Female	1.11 (0.81-1.53)	1.44 (1.13-1.84)**	1.09 (0.86-1.37)	0.89 (0.70-1.12)	1.16 (0.92-1.46)	1.02 (0.79-1.31)	0.93 (0.74-1.18)
Age group	18-34	1.11 (0.52-2.35)	1.21 (0.68-2.17)	1.07 (0.62-1.83)	1.12 (0.65-1.94)	0.99 (0.57-1.70)	1.24 (0.68-2.24)	2.08 (1.20-3.59)**
	35-44	1.21 (0.59-2.50)	1.39 (0.80-2.44)	1.09 (0.65-1.83)	1.41 (0.83-2.39)	0.96 (0.57-1.61)	1.29 (0.73-2.29)	1.93 (1.15-3.26)**
	45-54	1.09 (0.56-2.12)	0.97 (0.58-1.61)	0.96 (0.59-1.54)	1.18 (0.73-1.92)	0.86 (0.54-1.39)	1.25 (0.74-2.12)	1.40 (0.87-2.26)
	55-64	1.05 (0.58-1.90)	1.16 (0.73-1.84)	1.21 (0.79-1.85)	1.39 (0.90-2.14)	1.06 (0.69-1.62)	1.42 (0.88-2.28)	1.53 (1.00-2.35)*
	≥65	REF	REF	REF	REF	REF	REF	REF
Socioeconomic position	AB	REF	REF	REF	REF	REF	REF	REF
	C1C2	1.48 (1.06-2.07)*	1.42 (1.09-1.84)**	1.22 (0.95-1.57)	1.50 (1.16-1.95)**	1.31 (1.02-1.68)*	1.25 (0.95-1.64)	1.39 (1.08-1.80)**
	DE	1.30 (0.84-2.02)	1.67 (1.17-2.38)**	1.36 (0.97-1.88)	1.54 (1.10-2.16)**	1.41 (1.01-1.96)*	1.28 (0.90-1.84)	1.47 (1.05-2.05)*
Children in the household	No	REF	REF	REF	REF	REF	REF	REF
	Yes	0.72 (0.50-1.04)	0.78 (0.59-1.05)	0.82 (0.63-1.08)	1.07 (0.81-1.40)	0.91 (0.69-1.20)	0.79 (0.59-1.06)	0.77 (0.59-1.02)
Working status	Full-time	1.05 (0.66-1.66)	1.13 (0.78-1.63)	1.17 (0.81-1.59)	1.17 (0.83-1.65)	1.40 (1.00-1.97)*	1.12 (0.77-1.63)	1.22 (0.87-1.72)
	Part-time	1.00 (0.60-1.69)	0.96 (0.64-1.45)	0.97 (0.66-1.42)	1.01 (0.69-1.50)	1.04 (0.71-1.53)	0.96 (0.63-1.46)	0.93 (0.63-1.37)
	Self-employed	1.13 (0.60-2.12)	0.80 (0.50-1.29)	0.88 (0.56-1.38)	0.89 (0.56-1.40)	1.09 (0.70-1.70)	1.18 (0.72-1.95)	0.84 (0.54-1.32)
	Retired	1.06 (0.53-2.11)	1.24 (0.72-2.13)	1.06 (0.64-1.73)	1.33 (0.81-2.20)	1.19 (0.72-1.96)	1.13 (0.65-1.96)	1.16 (0.70-1.91)
	Not looking/ unable to work	REF	REF	REF	REF	REF	REF	REF
	Other	1.13 (0.37-3.49)	0.80 (0.36-1.80)	1.18 (0.54-2.57)	1.27 (0.58-2.76)	1.30 (0.60-2.84)	0.70 (0.31-1.57)	1.00 (0.45-2.18)
Region	London	REF	REF	REF	REF	REF	REF	REF
	North	1.26 (0.95-1.68)	1.10 (0.88-1.37)	1.27 (1.03-1.56)*	1.05 (0.86-1.29)	0.91 (0.74-1.12)	1.09 (0.87-1.36)	1.39 (1.13-1.71)**

Models adjusted for sex, age group, social class, children in the household, working status and region
* P<0.05, ** P<0.01, *** P<0.001

For peer review only - http://bmjopen.bmj.com/site/about/guidelines.xhtml

Table 3. Sociodemographic correlates of advertising exposure stratified by advertising setting (*n*=1552)

Sociodemographic characteristic		Traditional	Digital	Recreational	Functional	Transport
Sex	Male	REF	REF	REF	REF	REF
	Female	1.19 (0.92-1.54)	0.80 (0.63-1.02)	0.94 (0.71-1.26)	0.96 (0.76-1.21)	0.89 (0.70-1.14)
Age group	18-34	0.64 (0.34-1.18)	3.93 (2.18-7.08)***	1.37 (0.70-2.70)	1.27 (0.74-2.18)	1.96 (1.11-3.48)*
	35-44	0.74 (0.41-1.34)	3.06 (1.74-5.40)***	1.27 (0.66-2.44)	1.12 (0.67-1.88)	1.50 (0.87-2.61)
	45-54	0.77 (0.44-1.34)	1.69 (0.99-2.87)	0.84 (0.46-1.55)	0.89 (0.56-1.43)	0.99 (0.59-1.65)
	55-64	0.94 (0.57-1.54)	1.38 (0.85-2.24)	1.13 (0.66-1.94)	0.99 (0.65-1.51)	0.94 (0.59-1.49)
	≥65	REF	REF	REF	REF	REF
Socioeconomic position	AB	REF	REF	REF	REF	REF
	C1C2	1.52 (1.15-2.00)**	1.22 (0.93-1.60)	1.01 (0.73-1.39)	1.16 (0.90-1.49)	1.08 (0.82-1.41)
	DE	1.44 (1.00-2.08)*	1.50 (1.06-2.14)*	1.04 (0.68-1.59)	1.18 (0.85-1.65)	1.12 (0.78-1.59)
Children in the household	No	REF	REF	REF	REF	REF
	Yes	0.93 (0.69-1.26)	0.96 (0.72-1.27)	0.74 (0.52-1.05)	0.88 (0.67-1.15)	0.79 (0.60-1.06)
Working status	Full-time	1.01 (0.68-1.48)	1.28 (0.90-1.82)	1.18 (0.76-1.82)	1.07 (0.76-1.49)	1.50 (1.04-2.17)*
	Part-time	0.85 (0.55-1.31)	1.08 (0.73-1.61)	1.05 (0.64-1.73)	0.84 (0.57-1.23)	1.12 (0.74-1.71)
	Self-employed	0.80 (0.49-1.33)	0.93 (0.58-1.48)	0.87 (0.48-1.58)	0.95 (0.61-1.48)	1.55 (0.97-2.49)
	Retired	0.90 (0.50-1.60)	0.84 (0.49-1.45)	0.93 (0.49-1.76)	0.80 (0.49-1.32)	1.02 (0.59-1.76)
	Not looking/unable to work	REF	REF	REF	REF	REF
	Other	0.76 (0.32-1.78)	0.58 (0.25-1.36)	1.61 (0.66-3.94)	0.85 (0.39-1.84)	2.18 (0.98-4.86)
Region	London	REF	REF	REF	REF	REF
	North	1.13 (0.90-1.43)	1.20 (0.97-1.49)	1.11 (0.86-1.45)	1.16 (0.94-1.42)	2.05 (1.65-2.54)***

Models adjusted for sex, age group, social class, children in the household, working status and region
* P<0.05, ** P<0.01, *** P<0.001

For peer review only

Table 4. Weight status by exposure to advertising for less healthy foods and drinks (*n*=1552)

**Type of advertising**		**Underweight,** OR (95% CI)	**Overweight,** OR (95% CI)	**Obesity,** OR (95% CI)	**Missing,** OR (95% CI)
Any advertising exposure		0.94 (0.35-2.57)	1.08 (0.75-1.57)	1.08 (0.72-1.60)	0.94 (0.63-1.41)
By product category	Processed HFSS foods	1.31 (0.56-3.04)	1.17 (0.87-1.56)	1.12 (0.82-1.53)	0.89 (0.65-1.21)
	Sugary drinks	1.07 (0.51-2.24)	1.09 (0.84-1.43)	1.00 (0.75-1.33)	1.04 (0.78-1.41)
	Sugary cereals	1.03 (0.49-2.20)	1.21 (0.92-1.59)	1.29 (0.97-1.72)	1.03 (0.76-1.40)
	Sweet snacks	1.14 (0.54-2.39)	1.03 (0.79-1.35)	1.11 (0.83-1.47)	0.79 (0.58-1.06)
	Fast food	0.69 (0.32-1.47)	1.18 (0.88-1.58)	1.31 (0.95-1.80)	1.00 (0.72-1.38)
Digital food delivery services		0.71 (0.33-1.49)	1.22 (0.93-1.60)	1.40 (1.05-1.88)*	1.05 (0.78-1.43)
By advertising setting	Traditional	0.88 (0.40-1.94)	1.32 (0.98-1.79)	1.28 (0.93-1.77)	1.24 (0.89-1.74)
	Digital	1.12 (0.51-2.47)	1.28 (0.96-1.71)	1.80 (1.33-2.44)***	1.12 (0.82-1.54)
	Recreational	1.05 (0.41-2.66)	1.15 (0.81-1.63)	1.46 (1.02-2.09)*	1.07 (0.72-1.58)
	Functional	0.65 (0.31-1.38)	1.01 (0.78-1.32)	1.15 (0.86-1.52)	1.06 (0.79-1.43)
	Transport	0.63 (0.28-1.41)	1.07 (0.80-1.42)	1.20 (0.89-1.63)	0.78 (0.57-1.08)
Model adjusted for sex, age group, social class, children in the household, working status and region<br>* P<0.05, ** P<0.01, *** P<0.001					

For peer review only - http://bmjopen.bmj.com/site/about/guidelines.xhtml

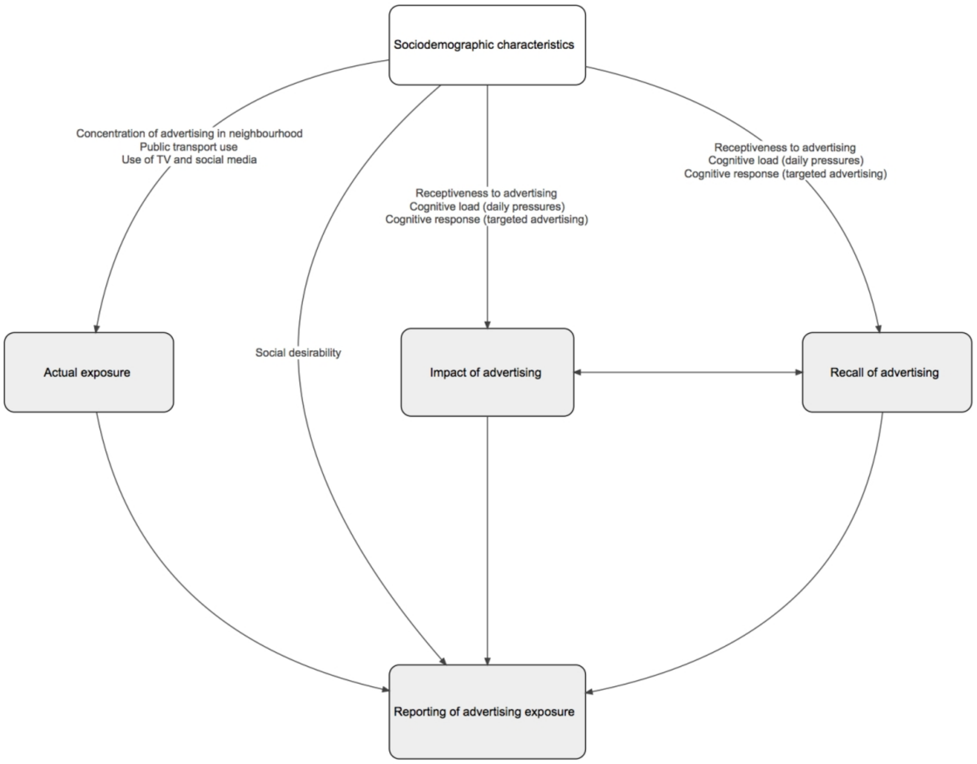

Potential explanations for sociodemographic differences in exposure to advertising for less healthy foods and drinks

525x414mm (72 x 72 DPI)

SUPPLEMENTARY MATERIAL

Supplementary Table S1. Definitions of product categories as presented in survey questions

**Product category**	**Definition in survey**
Processed HFSS foods	Processed foods high in salt, sugar and fat are those such as sugary drinks, meals from fast food chains, ready meals, sit down meals, sugary breakfast cereals, sweet snacks (e.g. chocolate bars, sweets, cookies/biscuits), savoury snacks (e.g. crisps, salted/flavoured nuts) and desserts (cakes, ice-cream and flavoured yoghurts).
Sugary drinks	SUGARY DRINKS are drinks that contain added sugar, like fizzy drinks, fruit drinks, sports drinks, energy drinks, diluted cordials/squash, chocolate milk, and specialty flavoured hot drinks that have added sugar but DO NOT INCLUDE pure fruit juice.
Sugary cereals	SUGARY CEREALS are those that contain added sugar such as chocolate or other flavouring/ fillings e.g. Coco Pops, Krave or are sweetened with frosting or added sugar e.g. Frosties, Sugar Puffs. DO NOT INCLUDE cereals like Weetabix, Ready Brek, Oatmeal/Porridge).
Sweet snacks	SWEET SNACKS are foods such as chocolate bars, cookies/biscuits, danish pastries, granola bars, sweets or other confectionary.
Fast food	These can be MEALS, FOODS OR DRINKS from fast-food chains or take-aways (e.g., Burger King, Dominos, McDonalds, KFC, fish and chip shops, Indian or Chinese Takeaway).

For peer review only

Supplementary Table S2. Number (%) of participants who reported seeing or hearing advertising for less healthy products and digital food delivery services in the past 7 days by sociodemographic characteristics, stratified by HFSS product category (*n*=1552)

Sociodemographic characteristic		Any	Processed HFSS foods	Sugary drinks	Sugary cereals	Sweet snacks	Fast food	Digital food delivery services
All		1314 (84.7)	1059 (68.2)	813 (52.4)	653 (42.1)	853 (55.0)	1107 (71.3)	852 (54.9)
Sex, N (%)	Male	370 (83.9)	277 (62.8)	228 (51.7)	195 (44.2)	235 (53.3)	316 (71.7)	249 (56.5)
	Female	944 (85.0)	782 (70.4)	585 (52.7)	458 (41.2)	618 (55.6)	791 (71.2)	603 (54.3)
X^2 (P-value)		0.28 (0.598)	8.36 (0.004)**	0.11 (0.734)	1.16 (0.281)	0.70 (0.404)	0.03 (0.857)	0.61 (0.435)
Age group (years), N (%)	18-34	157 (83.5)	129 (68.6)	96 (51.1)	74 (36.4)	105 (55.9)	131 (69.7)	113 (60.1)
	35-44	253 (84.6)	211 (70.6)	155(51.8)	134 (44.8)	161 (53.9)	210 (70.2)	176 (58.9)
	45-54	348 (84.7)	263 (64.0)	206 (50.1)	166 (40.4)	216 (52.6)	294 (71.5)	220 (53.5)
	55-64	286 (85.4)	235 (70.2)	190 (56.7)	151 (45.1)	193 (57.6)	252 (75.2)	191 (57.0)
	≥65	270 (84.6)	221 (69.3)	166 (52.0)	128 (40.1)	178 (55.8)	220 (69.0)	152 (47.7)
X^2 (P-value)		0.32 (0.988)	4.91 (0.297)	3.55 (0.471)	3.70 (0.448)	2.21 (0.697)	3.79 (0.435)	11.6 (0.020)*
Socioeconomic position, N (%)	AB	276 (80.9)	210 (61.6)	165 (48.4)	119 (34.9)	169 (49.6)	231 (67.7)	168 (49.3)
	C1C2	798 (86.2)	643 (69.4)	492 (53.1)	410 (44.3)	523 (56.5)	671 (72.5)	525 (56.7)
	DE	240 (84.2)	206 (72.3)	156 (54.7)	124 (43.5)	161 (56.5)	205 (71.9)	159 (55.8)
X^2 (P-value)		5.32 (0.070)	9.73 (0.008)*	3.02 (0.220)	9.29 (0.010)*	5.15 (0.076)	2.78 (0.249)	5.67 (0.059)
Children in the household, N (%)	No	951 (85.7)	768 (69.2)	598 (53.9)	465 (41.9)	621 (56.0)	807 (72.7)	615 (55.4)
	Yes	363 (82.1)	291 (65.8)	215 (48.6)	188 (42.5)	232 (52.5)	300 (67.9)	237 (53.6)
X^2 (P-value)		3.07 (0.080)	1.64 (0.200)	3.47 (0.063)	0.05 (0.817)	1.53 (0.217)	3.61 (0.058)	0.41 (0.523)
Working status, N (%)	Full-time	519 (84.8)	418 (68.3)	328 (53.6)	267 (43.6)	350 (57.2)	445 (72.7)	365 (59.6)
	Part-time	187 (83.9)	151 (67.7)	112 (50.2)	91 (40.8)	116 (52.0)	154 (69.1)	115 (51.6)
	Self-employed	113 (86.3)	82 (62.6)	64 (48.9)	49 (37.4)	68 (51.9)	97 (74.1)	67 (51.2)
	Retired	290 (84.8)	241 (70.5)	181 (52.9)	145 (42.4)	192 (56.1)	241 (70.5)	171 (50.0)
	Not looking/ unable to work	179 (83.6)	148 (69.2)	111 (51.9)	87 (40.7)	110 (51.4)	151 (70.6)	117 (54.7)
	Other	26 (86.7)	19 (63.3)	17 (56.7)	14 (46.7)	17 (56.7)	19 (63.3)	17 (56.7)
X^2 (P-value)		0.65 (0.986)	3.15 (0.676)	1.71 (0.887)	2.38 (0.795)	3.82 (0.575)	2.73 (0.741)	10.66 (0.059)
Region, N (%)	London	706 (83.4)	485 (68.8)	390 (55.3)	300 (42.6)	377 (53.5)	509 (72.2)	416 (59.0)
	North	608 (86.2)	574 (67.8)	423 (49.9)	353 (41.7)	476 (56.2)	598 (70.6)	436 (51.5)
X^2 (P-value)		2.47 (0.116)	0.19 (0.666)	4.46 (0.035)*	0.12 (0.728)	1.15 (0.283)	0.48 (0.489)	8.81 (0.003)**
* P<0.05, ** P<0.01, *** P<0.001								

For peer review only

Supplementary Table S3. Number (%) of participants who reported seeing or hearing advertising for less healthy products and digital food delivery services in the past 7 days by sociodemographic characteristics, stratified by advertising setting (*n*=1552)

Sociodemographic characteristic		Traditional	Digital	Recreational	Functional	Transport
All		1148 (74.0)	600 (38.7)	292 (18.8)	799 (51.5)	565 (36.4)
Sex, N (%)	Male	318 (72.11)	179 (40.6)	87 (19.7)	231 (52.4)	172 (39.0)
	Female	830 (74.7)	421 (37.9)	205 (18.5)	568 (51.1)	393 (35.4)
X^2 (P-value)		1.11 (0.293)	0.97 (0.325)	0.34 (0.562)	0.20 (0.655)	1.80 (0.180)
Age group (years), N (%)	18-34	130 (69.2)	108 (57.5)	41 (21.8)	107 (56.9)	87 (46.3)
	35-44	215 (71.9)	155 (51.8)	61 (20.4)	161 (53.9)	128 (42.8)
	45-54	300 (73.0)	157 (38.2)	66 (16.1)	204 (49.6)	144 (35.0)
	55-64	258 (77.0)	109 (32.5)	69 (20.6)	173 (51.6)	109 (32.5)
	≥65	245 (76.8)	71 (22.3)	55 (17.2)	154 (48.3)	97 (30.4)
X^2 (P-value)		6.08 (0.193)	91.4 (<0.001)***	4.85 (0.303)	4.77 (0.312)	20.66 (<0.001)***
Socioeconomic position, N (%)	AB	231 (67.7)	121 (35.5)	64 (18.8)	167 (49.0)	125 (36.7)
	C1C2	703 (75.9)	363 (39.2)	175 (18.9)	484 (52.3)	342 (36.9)
	DE	214 (75.1)	116 (40.7)	53 (18.6)	148 (51.9)	98 (34.4)
X^2 (P-value)		8.88 (0.012)**	2.07 (0.356)	0.01 (0.993)	1.11 (0.574)	0.62 (0.732)
Children in the household, N (%)	No	834 (75.1)	391 (35.2)	217 (19.6)	573 (51.6)	401 (36.1)
	Yes	314 (71.0)	209 (47.3)	75 (17.0)	226 (51.1)	164 (37.1)
X^2 (P-value)		2.75 (0.097)	19.39 (<0.001)***	1.38 (0.240)	0.03 (0.862)	0.13 (0.718)
Working status, N (%)	Full-time	451 (73.7)	284 (46.4)	126 (20.6)	333 (54.4)	254 (41.5)
	Part-time	161 (72.2)	91 (40.8)	40 (17.9)	107 (48.0)	72 (32.3)
	Self-employed	93 (71.0)	50 (38.2)	21 (16.0)	68 (51.9)	57 (43.5)
	Retired	262 (76.6)	78 (22.8)	59 (17.3)	163 (47.7)	100 (29.2)
	Not looking/ unable to work	160 (74.8)	88 (41.1)	38 (17.8)	113 (52.8)	66 (30.8)
	Other	21 (70.0)	9 (30.0)	8 (26.7)	15 (50.0)	16 (53.3)
X^2 (P-value)		2.54 (0.770)	53.67 (<0.001)***	3.95 (0.556)	5.38 (0.371)	25.52 (<0.001)***
Region, N (%)	London	528 (75.9)	287 (40.7)	139 (19.7)	376 (53.3)	321 (45.5)
	North	620 (73.2)	313 (37.0)	153 (18.1)	423 (49.9)	244 (28.8)
X^2 (P-value)		0.57 (0.449)	2.29 (0.130)	0.69 (0.407)	1.77 (0.183)	46.48 (<0.001)***
* P<0.05, ** P<0.01, *** P<0.001						

For peer review only

Supplementary Table S4. Number (%) of participants who reported seeing or hearing advertising for less healthy products and digital food delivery services in the past 7 days by product/service advertised and advertising setting, stratified by region (*n*=1552)

**Advertised product/service**	**Region**	All	Traditional	Digital	Recreational	Functional	Transport
All, N (%)	All	1314 (84.7)	1148 (74.0)	600 (38.7)	292 (18.8)	799 (51.5)	565 (36.4)
	London	608 (86.2)	528 (74.9)	287 (40.7)	139 (19.7)	376 (53.3)	321 (45.5)
	North	706 (83.4)	620 (73.2)	313 (37.0)	153 (18.1)	423 (49.9)	244 (28.8)
X^2 (P-value)		2.47 (0.116)	0.57 (0.449)	2.29 (0.130)	0.69 (0.407)	1.77 (0.183)	46.48 (<0.001)***
Processed HFSS foods, N (%)	All	1059 (68.2)	853 (55.0)	384 (24.7)	135 (8.7)	547 (35.2)	376 (24.2)
	London	485 (68.8)	387 (54.9)	177 (25.1)	59 (8.4)	253 (35.9)	223 (31.6)
	North	574 (67.8)	466 (55.0)	207 (24.4)	76 (9.0)	294 (34.7)	153 (18.1)
X^2 (P-value)		0.19 (0.666)	0.002 (0.961)	0.09 (0.762)	0.18 (0.674)	0.23 (0.629)	38.58 (<0.001)***
Sugary drinks, N (%)	All	813 (52.4)	596 (38.3)	223 (14.4)	114 (7.4)	318 (20.5)	206 (13.3)
	London	390 (55.3)	279 (39.6)	105 (14.9)	56 (7.9)	155 (22.0)	117 (16.6)
	North	423 (49.9)	316 (37.3)	118 (13.9)	58 (6.9)	163 (19.2)	89 (10.5)
X^2 (P-value)		4.46 (0.035)*	0.84 (0.361)	0.29 (0.591)	0.68 (0.410)	1.77 (0.183)	12.39 (<0.001)***
Sugary cereals, N (%)	All	653 (42.1)	502 (32.4)	123 (7.9)	48 (3.1)	215 (13.9)	75 (4.8)
	London	300 (42.6)	230 (32.6)	62 (8.8)	26 (3.7)	100 (14.2)	56 (7.9)
	North	353 (41.7)	272 (32.1)	61 (7.2)	22 (2.6)	115 (13.6)	19 (2.2)
X^2 (P-value)		0.12 (0.728)	0.05 (0.830)	1.33 (0.248)	1.53 (0.217)	0.12 (0.730)	27.18 (<0.001)***
Sweet snacks, N (%)	All	853 (55.0)	639 (41.2)	218 (14.1)	101 (6.5)	339 (21.8)	171 (11.0)
	London	377 (53.5)	273 (38.7)	101 (14.3)	45 (6.4)	163 (23.1)	109 (15.5)
	North	476 (56.2)	366 (43.2)	117 (13.8)	56 (6.6)	176 (20.8)	62 (7.3)
X^2 (P-value)		1.15 (0.283)	3.20 (0.074)	0.08 (0.772)	0.03 (0.856)	1.24 (0.266)	26.01 (<0.001)***
Fast food, N (%)	All	1107 (71.3)	855 (55.1)	312 (20.1)	103 (6.6)	380 (24.5)	327 (21.1)
	London	509 (72.2)	386 (54.8)	137 (19.4)	53 (7.5)	179 (25.4)	198 (28.1)
	North	598 (70.6)	469 (55.4)	175 (20.7)	50 (5.9)	201 (23.7)	129 (15.2)
X^2 (P-value)		0.48 (0.489)	0.06 (0.807)	0.36 (0.548)	1.62 (0.203)	0.57 (0.449)	38.23 (<0.001)***
Digital food delivery services, N (%)	All	852 (54.9)	647 (41.7)	297 (19.1)	37 (2.4)	115 (7.4)	126 (8.1)
	London	416 (59.0)	299 (42.4)	163 (23.1)	34 (3.4)	66 (9.4)	96 (13.6)
	North	436 (51.5)	348 (41.1)	134 (15.8)	13 (1.5)	49 (5.8)	30 (3.5)
X^2 (P-value)		8.81 (0.003)**	0.28 (0.598)	13.25 (<0.001)**	5.78 (0.016)*	7.17 (0.007)*	52.36 (<0.001)***
* P<0.05, ** P<0.01, *** P<0.001							

For peer review only

Supplementary Table S5. Interaction terms (P-values) for interactions between sociodemographic characteristics and region on self-reported advertising exposure

Advertising type	Sex##Region	Age group##Region	Socioeconomic position##Region	Children##Region	Working status##Region
Any advertising	1.52 (0.677)	3.91 (0.917)	5.81 (0.325)	4.23 (0.238)	2.83 (0.993)
Processed HFSS foods	10.64 (0.014)**	7.35 (0.600)	9.64 (0.086)	3.41 (0.332)	6.15 (0.863)
Sugary drinks	2.31 (0.511)	6.40 (0.700)	6.62 (0.251)	3.45 (0.327)	4.59 (0.949)
Sugary cereal	1.96 (0.580)	5.76 (0.764)	13.08 (0.023)*	2.32 (0.508)	14.30 (0.217)
Sweet snacks	2.60 (0.457)	3.83 (0.922)	8.88 (0.114)	1.47 (0.690)	9.19 (0.605)
Fast food	0.08 (0.995)	4.05 (0.908)	3.37 (0.643)	3.48 (0.323)	4.28 (0.961)
Digital food delivery services	4.27 (0.233)	16.41 (0.059)	8.56 (0.128)	4.15 (0.245)	13.14 (0.284)
Traditional advertising	2.26 (0.520)	7.82 (0.553)	11.37 (0.044)*	1.39 (0.709)	9.29 (0.595)
Digital advertising	5.79 (0.122)	47.08 (<0.001)***	9.82 (0.081)	3.27 (0.352)	22.98 (0.018)*
Advertising in recreational environments	2.32 (0.508)	10.03 (0.348)	3.90 (0.564)	5.17 (0.160)	5.90 (0.880)
Advertising in functional environments	1.60 (0.660)	9.93 (0.357)	3.81 (0.577)	3.20 (0.362)	9.51 (0.575)
Advertising across transport networks	5.31 (0.151)	29.90 (<0.001)***	7.43 (0.190)	7.25 (0.065)	16.69 (0.117)
* P<0.05, ** P<0.01, *** P<0.001					

For peer review only

Supplementary Table S6. Sociodemographic correlates of advertising exposure stratified by product/service advertised and region where interactions were significant (*n*=1552)

Product/service advertised	Sex	Age group	Socioeconomic position	Children in the household	Working status
Any advertising	No interaction	No interaction	No interaction	No interaction	No interaction
Processed HFSS foods	London<br>1.18 (0.82-1.70)<br>North of England<br>1.70 (1.22-2.37)**	No interaction	No interaction	No interaction	No interaction
Sugary drinks	No interaction	No interaction	No interaction	No interaction	No interaction
Sugary cereal	No interaction	No interaction	London<br>C1C2: 1.35 (0.94-1.95)<br>DE: 1.75 (1.06-2.88)*<br>North of England<br>C1C2: 1.62 (1.11-2.36)*<br>DE: 1.41 (0.88-2.25)	No interaction	No interaction
Sweet snacks	No interaction	No interaction	No interaction	No interaction	No interaction
Fast food	No interaction	No interaction	No interaction	No interaction	No interaction
Digital food delivery services	No interaction	No interaction	No interaction	No interaction	No interaction

For peer review only

Supplementary Table S7. Sociodemographic correlates of advertising exposure stratified by advertising setting and region where interactions were significant (*n*=1552)

Advertising setting	Sex	Age group	Socioeconomic position	Children in the household	Working status
Traditional	No interaction	No interaction	London<br>C1C2: 1.41 (0.95-2.10)<br>DE: 1.90 (1.06-3.41)*<br>North of England<br>C1C2: 1.53 (1.04-2.25)*<br>DE: 1.17 (0.72-1.91)	No interaction	No interaction
Digital	No interaction	London<br>35-44: 0.84 (0.45-1.55)<br>45-54: 0.37 (0.20-0.68)**<br>55-64: 0.27 (0.14-0.52)***<br>≥65: 0.15 (0.06-0.37)***<br>North of England<br>35-44: 0.72 (0.44-1.18)<br>45-54: 0.46 (0.28-0.74)**<br>55-64: 0.42 (0.25-0.71)***<br>≥65: 0.37 (0.17-0.82)***	No interaction	No interaction	London<br>Part-time: 0.69 (0.40-1.16)<br>Self-employed: 0.69 (0.40-1.17)<br>Retired: 0.64 (0.31-1.35)<br>At home: 0.50 (0.29-0.88)*<br>Other: 0.08 (0.02-0.37)**<br>North of England<br>Part-time: 0.99 (0.64-1.55)<br>Self-employed: 0.71 (0.38-1.33)<br>Retired: 0.67 (0.34-1.32)<br>At home: 1.06 (0.67-1.66)<br>Other: 2.67 (0.75-9.55)
Recreational	No interaction	No interaction	No interaction	No interaction	No interaction
Functional	No interaction	No interaction	No interaction	No interaction	No interaction
Transport	No interaction	London<br>35-44: 0.72 (0.39-1.30)<br>45-54: 0.52 (0.29-0.93)*<br>55-64: 0.55 (0.29-1.04)*<br>≥65: 0.73 (0.32-1.68)<br>North of England<br>35-44: 0.86 (0.52-1.43)<br>45-54: 0.49 (0.30-0.82)**<br>55-64: 0.44 (0.25-0.77)**<br>≥65: 0.34 (0.15-0.78)**	No interaction	No interaction	No interaction

For peer review only

STROBE Statement—checklist of items that should be included in reports of observational studies

	Item No.	Recommendation	Page No.	Relevant text from manuscript
**Title and abstract**	1	(*a*) Indicate the study's design with a commonly used term in the title or the abstract	1	Cross-sectional analysis
		(*b*) Provide in the abstract an informative and balanced summary of what was done and what was found	2-3	
**Introduction**				
Background/rationale	2	Explain the scientific background and rationale for the investigation being reported	5-6	
Objectives	3	State specific objectives, including any prespecified hypotheses	7	
**Methods**				
Study design	4	Present key elements of study design early in the paper	7-11	
Setting	5	Describe the setting, locations, and relevant dates, including periods of recruitment, exposure, follow-up, and data collection	7-11	
Participants	6	(*a*) *Cohort study*—Give the eligibility criteria, and the sources and methods of selection of participants. Describe methods of follow-up<br>*Case-control study*—Give the eligibility criteria, and the sources and methods of case ascertainment and control selection. Give the rationale for the choice of cases and controls<br>*Cross-sectional study*—Give the eligibility criteria, and the sources and methods of selection of participants	7	
		(*b*) *Cohort study*—For matched studies, give matching criteria and number of exposed and unexposed<br>*Case-control study*—For matched studies, give matching criteria and the number of controls per case	N/A	
Variables	7	Clearly define all outcomes, exposures, predictors, potential confounders, and effect modifiers. Give diagnostic criteria, if applicable	8-10	
Data sources/ measurement	8*	For each variable of interest, give sources of data and details of methods of assessment (measurement). Describe comparability of assessment methods if there is more than one group	*8-10*	
Bias	9	Describe any efforts to address potential sources of bias	N/A	
Study size	10	Explain how the study size was arrived at	7	

Continued on next page

For peer review only - http://bmjopen.bmj.com/site/about/guidelines.xhtml

For peer review only

Quantitative variables	11	Explain how quantitative variables were handled in the analyses. If applicable, describe which groupings were chosen and why	8-10
Statistical methods	12	(*a*) Describe all statistical methods, including those used to control for confounding	11
		(*b*) Describe any methods used to examine subgroups and interactions	11
		(*c*) Explain how missing data were addressed	8-11
		(*d*) *Cohort study*—If applicable, explain how loss to follow-up was addressed<br>*Case-control study*—If applicable, explain how matching of cases and controls was addressed<br>*Cross-sectional study*—If applicable, describe analytical methods taking account of sampling strategy	N/A
		(*e*) Describe any sensitivity analyses	N/A
**Results**			
Participants	13*	(a) Report numbers of individuals at each stage of study—eg numbers potentially eligible, examined for eligibility, confirmed eligible, included in the study, completing follow-up, and analysed	11
		(b) Give reasons for non-participation at each stage	10
		(c) Consider use of a flow diagram	
Descriptive data	14*	(a) Give characteristics of study participants (eg demographic, clinical, social) and information on exposures and potential confounders	11
		(b) Indicate number of participants with missing data for each variable of interest	10
		(c) *Cohort study*—Summarise follow-up time (eg, average and total amount)	
Outcome data	15*	*Cohort study*—Report numbers of outcome events or summary measures over time	
		Case-control study—Report numbers in each exposure category, or summary measures of exposure	
		Cross-sectional study—Report numbers of outcome events or summary measures	*11*
Main results	16	(*a*) Give unadjusted estimates and, if applicable, confounder-adjusted estimates and their precision (eg, 95% confidence interval). Make clear which confounders were adjusted for and why they were included	12-15
		(*b*) Report category boundaries when continuous variables were categorized	8-11
		(*c*) If relevant, consider translating estimates of relative risk into absolute risk for a meaningful time period	

Continued on next page

For peer review only - http://bmjopen.bmj.com/site/about/guidelines.xhtml

Other analyses	17	Report other analyses done—eg analyses of subgroups and interactions, and sensitivity analyses	
**Discussion**			
Key results	18	Summarise key results with reference to study objectives	15-16
Limitations	19	Discuss limitations of the study, taking into account sources of potential bias or imprecision. Discuss both direction and magnitude of any potential bias	16-17
Interpretation	20	Give a cautious overall interpretation of results considering objectives, limitations, multiplicity of analyses, results from similar studies, and other relevant evidence	17-20
Generalisability	21	Discuss the generalisability (external validity) of the study results	17
**Other information**			
Funding	22	Give the source of funding and the role of the funders for the present study and, if applicable, for the original study on which the present article is based	22-23

*Give information separately for cases and controls in case-control studies and, if applicable, for exposed and unexposed groups in cohort and cross-sectional studies.

Note: An Explanation and Elaboration article discusses each checklist item and gives methodological background and published examples of transparent reporting. The STROBE checklist is best used in conjunction with this article (freely available on the Web sites of PLoS Medicine at http://www.plosmedicine.org/, Annals of Internal Medicine at http://www.annals.org/, and Epidemiology at http://www.epidem.com/). Information on the STROBE Initiative is available at www.strobe-statement.org.

For peer review only

BMJ Open

Sociodemographic differences in self-reported exposure to high fat, salt and sugar food and drink advertising: a cross-sectional analysis of 2019 UK panel data

Journal:	*BMJ Open*
Manuscript ID	bmjopen-2020-048139.R1
Article Type:	Original research
Date Submitted by the Author:	16-Mar-2021

Complete List of Authors:	Yau, Amy; London School of Hygiene & Tropical Medicine, Public Health, Environments & Society<br>Adams, J; University of Cambridge, Centre for Diet & Activity Research<br>Boyland, Emma; University of Liverpool, Department of Psychology<br>Burgoine, Thomas; University of Cambridge, Centre for Diet and Activity Research<br>Cornelsen, Laura; London School of Hygiene & Tropical Medicine, Public Health, Environments & Society<br>De Vocht, Frank; University of Bristol, Population Health Sciences; National Institute for Health Research Applied Research Collaboration West<br>Egan, Matt; London School of Hygiene & Tropical Medicine, Public Health, Environments & Society<br>Er, Vanessa; London School of Hygiene & Tropical Medicine, Public Health, Environments & Society<br>Lake, Amelia; Teesside University, Centre for Public Health Research; Centre for Translational Research in Public Health, Fuse<br>Lock, Karen; London School of Hygiene & Tropical Medicine, Health Services Research & Policy<br>Mytton, Oliver; University of Cambridge, Centre for Diet and Activity Research<br>Petticrew, Mark; London School of Hygiene & Tropical Medicine, Public Health, Environments & Society<br>Thompson, Claire; University of Hertfordshire, Centre for Research in Public Health and Community Care<br>White, Martin; University of Cambridge, Centre for Diet and Activity Research<br>Cummins, Steven; London School of Hygiene & Tropical Medicine, Public Health, Environments & Society

**Primary Subject Heading**:	Public health
Secondary Subject Heading:	Public health
Keywords:	PUBLIC HEALTH, SOCIAL MEDICINE, NUTRITION & DIETETICS

For peer review only - http://bmjopen.bmj.com/site/about/guidelines.xhtml

I, the Submitting Author has the right to grant and does grant on behalf of all authors of the Work (as defined in the below author licence), an exclusive licence and/or a non-exclusive licence for contributions from authors who are: i) UK Crown employees; ii) where BMJ has agreed a CC-BY licence shall apply, and/or iii) in accordance with the terms applicable for US Federal Government officers or employees acting as part of their official duties; on a worldwide, perpetual, irrevocable, royalty-free basis to BMJ Publishing Group Ltd ("BMJ") its licensees and where the relevant Journal is co-owned by BMJ to the co-owners of the Journal, to publish the Work in this journal and any other BMJ products and to exploit all rights, as set out in our licence.

The Submitting Author accepts and understands that any supply made under these terms is made by BMJ to the Submitting Author unless you are acting as an employee on behalf of your employer or a postgraduate student of an affiliated institution which is paying any applicable article publishing charge ("APC") for Open Access articles. Where the Submitting Author wishes to make the Work available on an Open Access basis (and intends to pay the relevant APC), the terms of reuse of such Open Access shall be governed by a Creative Commons licence – details of these licences and which Creative Commons licence will apply to this Work are set out in our licence referred to above.

Other than as permitted in any relevant BMJ Author's Self Archiving Policies, I confirm this Work has not been accepted for publication elsewhere, is not being considered for publication elsewhere and does not duplicate material already published. I confirm all authors consent to publication of this Work and authorise the granting of this licence.

For peer review only

Sociodemographic differences in self-reported exposure to high fat, salt and sugar food and drink advertising: a cross-sectional analysis of 2019 UK panel data

Amy Yau[1], Jean Adams[2], Emma Boyland[3], Thomas Burgoine[2], Laura Cornelsen[1], Frank de Vocht[4,5], Matt Egan[6], Vanessa Er[1], Amelia A Lake[7,8], Karen Lock[9], Oliver Mytton[2], Mark Petticrew[6], Claire Thompson[10], Martin White[2], Steven Cummins[1]

1 Population Health Innovation Lab, Department of Public Health, Environments & Society, Faculty of Public Health & Policy, London School of Hygiene & Tropical Medicine, London, United Kingdom
2 Centre for Diet & Activity Research, MRC Epidemiology Unit, University of Cambridge, Cambridge, United Kingdom
3 Department of Psychology, Institute of Population Health, University of Liverpool, Liverpool, United Kingdom
4 Population Health Sciences, Bristol Medical School, University of Bristol, United Kingdom
5 National Institute for Health Research Applied Research Collaboration West (NIHR ARC West), Bristol, United Kingdom
6 Department of Public Health, Environments & Society, Faculty of Public Health & Policy, London School of Hygiene & Tropical Medicine, London, United Kingdom
7 Centre for Public Health Research, School of Health and Life Sciences, Teesside University, Middlesbrough, United Kingdom
8 Fuse, The Centre for Translational Research in Public Health, Newcastle upon Tyne, United Kingdom
9 Department of Health Services Research & Policy, Faculty of Public Health & Policy, London School of Hygiene & Tropical Medicine, London, United Kingdom
10 Centre for Research in Public Health and Community Care, School of Health and Social Work, University of Hertfordshire, Hatfield, United Kingdom

Corresponding author: Amy Yau (amy.yau@lshtm.ac.uk), London School of Hygiene & Tropical Medicine, 15-17 Tavistock Place, London WC1H 9SH

Word count: 4214

For peer review only

ABSTRACT

Objectives To explore sociodemographic differences in exposure to advertising for foods and drinks high in fat, salt and sugar (HFSS) and whether exposure is associated with body mass index (BMI).

Design Cross-sectional survey

Setting United Kingdom

Participants 1552 adults recruited to the Kantar Fast Moving Consumer Goods panel for London and the North of England

Outcome measures Self-reported advertising exposure stratified by product/service advertised (processed HFSS foods; sugary drinks; sugary cereals; sweet snacks; fast food or digital food delivery services) and advertising setting (traditional; digital; recreational; functional or transport); BMI and sociodemographic characteristics.

Results Overall, 84.7% of participants reported exposure to HFSS advertising in the past 7 days. Participants in the middle (vs high) socioeconomic group had higher odds of overall self-reported exposure (OR 1.48; 95% CI 1.06-2.07). Participants in the low (vs high) socioeconomic group had higher odds of reporting exposure to advertising for three of five product categories (ORs ranging from 1.36 to 1.67), advertising for digital food delivery services (OR 1.47; 95% CI 1.05-2.05), traditional advertising (OR 1.44; 95% CI 1.00-2.08) and digital advertising (OR 1.50; 95% CI 1.06-2.14). Younger adults (18-34 years vs ≥65 years)

For peer review only

had higher odds of reporting exposure to advertising for digital food delivery services (OR 2.08; 95% CI 1.20-3.59), digital advertising (OR 3.93; 95% CI 2.18-7.08) and advertising across transport networks (OR 1.96, 95% CI 1.11-3.48). Exposure to advertising for digital food delivery services (OR 1.40; 95% CI 1.05-1.88), digital advertising (OR 1.80; 95% CI 1.33-2.44) and advertising in recreational environments (OR 1.46; 95% CI 1.02-2.09) were associated with increased odds of obesity.

Conclusions Exposure to less healthy product advertising was prevalent, with adults in lower socioeconomic groups and younger adults more likely to report exposure. Broader restrictions may be needed to reduce sociodemographic differences in exposure to less healthy product advertising.

For peer review only

ARTICLE SUMMARY

Strengths and limitations of this study

- This study investigates exposure to advertising for a range of less healthy products in various settings, including some that have been less studied (e.g. advertising for digital food delivery services and advertising across transport networks)
- Self-reported exposure may not be an accurate reflection of actual exposure to advertising due to poor recall or social desirability bias
- The cross-sectional design of this study limits our interpretation of the findings as reverse causality may explain the associations we observed
- Participants were from two regions (London and the North of England), so may not be representative of the United Kingdom as a whole

For peer review only

INTRODUCTION

In recent years there has been an increased focus on the potential contribution of marketing and advertising of less healthy foods and drinks, such as those higher in fat, salt and sugar (HFSS), to population diet and diet-related disease.[1,2] Over £300 million was spent on advertising of less healthy foods and drinks in the UK in 2017.[3,4] This spend substantially outweighs that for healthier products. For example, £12 million was spent on advertising vegetables compared to almost £87 million on soft drinks in the UK in 2015.[4] Advertising and marketing of less healthy foods and drinks to children is widespread and is associated with children's preferences, requests for purchases, and consumption.[5–8] In adults, evidence is mixed, but some studies have found that exposure to less healthy food and drink advertising influences purchasing and consumption, and normalises the consumption of less healthy foods and drinks. [9–14] Advertising exposure may therefore be a plausible influence on obesity and diet-related non-communicable diseases.[15,16] A systematic review found that children from ethnic minority and socioeconomically disadvantaged backgrounds were disproportionately exposed to advertising for less healthy foods.[17] In adults, exposure to outdoor advertising and television advertising of less healthy foods are correlated with socioeconomic position in the UK.[18,19] These findings suggest that differential exposure to less healthy advertising may be contributing to the higher burden of obesity and diet-related non-communicable diseases among lower socioeconomic groups.[20–22]

In response, the regulation of food and drink marketing and advertising has been increasingly promoted as a policy lever for obesity prevention by local and national governments and the World Health Organisation.[8,23,24] In the UK, policies have focused

For peer review only - http://bmjopen.bmj.com/site/about/guidelines.xhtml

on highly-processed HFSS products as determined by the Nutrient Profiling Model.[23,25] However, restrictions on other advertising may also be needed. The use and advertising of digital food delivery services have increased dramatically in recent years.[26] These services often deliver takeaway fast food or restaurant meals, which tend to be less healthy than home-prepared food.[27–29] Therefore, regulating the promotion of digital food delivery services may also be a potential policy lever to reduce consumption of less healthy products.

A wide range of advertising channels are used by companies to promote their products and brand. The use of multiple advertising settings both reinforces the messaging and helps to reach a wider audience.[30] Outdoor advertising is thought to reach 98% of the UK population at least once a week and is especially effective at reaching young, urban, affluent consumers.[31] Advertising across the Transport for London network generated £152.1 million in 2017/18, accounting for 40% of London's outdoor advertising spend and 20% of outdoor advertising spend across the UK.[32] Digital advertising has grown, with online advertisers spending £13.4 billion in 2018 in the UK, and is set to grow further.[33,34] Digital advertising has the advantage of allowing more customisation and is often targeted towards younger people, especially those in more disadvantaged areas.[35–37] Advertising through more traditional media, such as television, remains popular with food companies and are considered cost-effective and wide-reaching.[33]

Obesity and diet quality are socially patterned, with greater prevalence of obesity and poorer diet quality associated with lower socioeconomic position.[21,38] Socioeconomic inequalities in exposure to advertising of less healthy foods and drinks may be a mechanism

For peer review only

For peer review only - http://bmjopen.bmj.com/site/about/guidelines.xhtml

for the generation of socioeconomic inequalities in diet and obesity. While there is some evidence that levels of exposure to outdoor advertising and television advertising for HFSS products differ by socioeconomic position, the evidence base for sociodemographic correlates of advertising exposure in the UK remains limited.[18,19] Very little research has been conducted to explore differences in exposure to a wider range of advertising settings, such as digital advertising and advertising across transport networks.[39] Exposure to advertising for digital food delivery services is also poorly understood. Further, few studies have examined sociodemographic differences in advertising exposure beyond socioeconomic position. There has also been limited work that directly explores associations between exposure to less healthy food and drink advertising and obesity. In this paper we address these gaps by using survey data from the UK (London and the North of England) to explore sociodemographic differences in: (i) overall exposure to less healthy food and drink advertising, (ii) advertising exposure stratified by product category, (iii) exposure to advertising for digital food delivery services and (iv) advertising exposure stratified by advertising setting. We then examine the association between advertising exposure and body mass index (BMI).

METHODS

Data

We used baseline cross-sectional data from a study evaluating the impact of restricting advertising of HFSS foods and drinks across the Transport for London network.[32,40] Data were from households recruited to the UK Kantar Fast Moving Consumer Goods (FMCG) panel for London and the North of England (*n*=1552 households). Our sample size was based on the maximum number of available households within the panel for the two regions. The

For peer review only - http://bmjopen.bmj.com/site/about/guidelines.xhtml

main food shopper from each recruited household was asked to complete an online survey (between 10th and 18th February 2019) on recent exposure to advertising of less healthy foods and drinks. The response rate was 71%. Households were recruited to the consumer panel through stratified random sampling and were representative of the regions from which they were sampled on the basis of household size, number of children, socioeconomic position and age of main shopper.[41,42] Panel households are recruited by Kantar through post and email.[42]

Ethical approval for this study was granted by the London School of Hygiene & Tropical Medicine Research Ethics Committee (ref no: 16297 /RR/11721).

Sociodemographic characteristics

Based on self-reported survey data, participants were categorised by sociodemographic characteristics: sex (male and female), age group (18-34 years, 35-44 years, 45-54 years, 55-64 years and ≥65 years), children <16 years in the household (yes or no), socioeconomic position and working status. For socioeconomic position, participants were classified according to the National Readership Survey (NRS) occupational social grade classification (A, B, C1, C2, D, E).[43] We categorised NRS social grade into three socioeconomic groups: high (AB), middle (C1C2) and low (DE). For working status, we categorised participants into six categories: full-time employee, part-time employee, self-employed, retired, not looking for work or unable to work (looking after home or family, long term sick or disabled, away from work due to illness, maternity leave, holiday or unemployed and not looking for work) or other (government sponsored training scheme, other paid work, student, actively looking for paid work or other).

For peer review only - http://bmjopen.bmj.com/site/about/guidelines.xhtml

Region

From household postcode data, participants were categorised as living in either London (Greater London) or the North of England (North West, North East or Yorkshire and the Humber).

Measuring exposure to advertising

We assessed exposure to advertising for five of the most commonly advertised food and drink product categories of current policy concern: processed HFSS foods, sugary drinks, sugary cereals, sweet snacks and fast food.[44] Definitions of the product categories used in the survey were adapted from the International Food Policy Study and are available in **Supplementary Table S1**.[45] In addition, exposure to advertising for digital food delivery services was assessed with a question on exposure to advertising for food delivery apps, with the market-leading services (Uber Eats, Deliveroo, Just Eat and FoodHub) listed as examples.

Survey questions were structured as follows: *"In the last 7 days, have you seen or heard advertisements for* [category] *in the following places?"*. Within each question, 19 places where advertisements may have been seen or heard were specified. Using methods adapted from Forde et al,[12] we re-categorised the 19 places into five advertising settings prior to analysis: traditional advertising, digital advertising, advertising in recreational environments, advertising in functional environments and advertising across transport networks (**Box 1**). Participants were also able to report other places using free text. We allocated these text responses into the five advertising settings, treating the categories as

For peer review only - http://bmjopen.bmj.com/site/about/guidelines.xhtml

mutually exclusive. Where there was ambiguity in categorisation, advertising setting was categorised based on *where* the advertisement was seen or heard rather than what medium was used. For example, a digital advertisement *at a bus stop* was categorised as transport.

Box 1. Categorisation of advertising setting

Advertising setting	Description	Included survey responses
Traditional	Physical, non-digital text and radio media and direct marketing	Television, radio, text message, newspaper/magazine, email and leaflet
Digital	Advertising seen or heard through digital platforms and social media	Online/internet, mobile app, video game and social media
Recreational	Advertising placed in environments that people interact with for enjoyment and leisure purposes	Film/cinema, leisure centre/gym/community centre, sports event/concert/community event, giveaway/sample/special offer and pub
Functional	Advertising placed in environments that people visit for a specific purpose and to complete a specific task (e.g. school, work and shops)	Billboard/outdoor signs, telephone boxes, school/college/university, signs or displays in supermarket/convenience stores/restaurants, delivery drivers, doctor's surgery, shopping centre and motorway services
Transport	Advertising placed in environments related to transport	Outside/inside buses, outside/inside tube, tram or train, outside/inside of tube or train station, bus stop, taxi and back of bus ticket

For peer review only

We coded advertising exposure into two categories: exposed or not. For overall exposure, participants who reported seeing or hearing advertisements for any of the six product/service categories (processed HFSS foods; sugary drinks; sugary cereals; sweet snacks; fast food and digital food delivery services) were classified as exposed.

BMI and weight status

BMI (weight/$height^2$) was calculated using self-reported height and weight data. BMI was available for 81.7% of participants (*n*=1268), who were then classified into four categories:

For peer review only - http://bmjopen.bmj.com/site/about/guidelines.xhtml

underweight (BMI<18.5), normal (BMI 18.5-25), overweight (BMI 25-30) and obese (BMI ≥30). A fifth category contained participants with missing BMI data (*n*=284).

Statistical methods

We calculated the number (%) of participants reporting exposure to less healthy food and drink advertising (including digital food delivery services) in the past 7 days, overall and stratified by product category and advertising setting. Using logistic regression models we estimated odds ratios, with 95% confidence intervals (CIs), for the association between sociodemographic characteristics and advertising exposure. As advertising exposure may be influenced by social and environmental factors, independently or in combination, we tested for interactions between sociodemographic characteristics and region (London or the North of England). Separate models were used to look at overall advertising exposure, advertising exposure stratified by product category, exposure to advertising for digital food delivery services and exposure stratified by advertising setting. We also used logistic regression models to investigate the association between self-reported advertising exposure and odds of living with overweight or obesity. All regression models were mutually adjusted for sex, age group, socioeconomic position, children in the household, working status and region. All analyses were conducted in Stata IC version 16 and completed in September 2020.

Patient and public involvement

Patients and the public were not involved in the design, or conduct, or reporting, or dissemination plans of our research.

RESULTS

For peer review only - http://bmjopen.bmj.com/site/about/guidelines.xhtml

Overall, 1552 participants were included in this study (**Table 1**) with 45.4% of participants living in London and 54.6% in the North of England. The majority of participants were female (71.6%), in the middle socioeconomic group (C1C2) (59.7%), had no children <16 years in the household (71.5%) and were in work (64.1%).

Self-reported exposure to advertising

Overall, 84.7% of participants reported seeing or hearing advertising for less healthy foods and drinks and/or digital food delivery services in the past 7 days (see **Supplementary Table S2**). The proportion of participants reporting exposure to advertising differed according to product category: 68.2% for processed HFSS foods, 52.4% for sugary drinks, 42.1% for sugary cereals, 55.0% for sweet snacks, 71.3% for fast food and 54.9% for digital food delivery services. Reported exposure also varied according to advertising setting: 74.0% of participants reported seeing or hearing traditional advertising, 38.7% digital advertising, 18.8% advertising in recreational environments, 51.5% advertising in functional environments and 36.4% advertising across transport networks (see **Supplementary Table S3**). For most product categories, reported exposure was most common through traditional advertising, followed by advertising in functional environments, digital advertising, advertising across transport networks, and lastly advertising in recreational environments. However, for digital food delivery services, digital was the second highest reported setting after traditional. Self-reported exposure to advertising was higher in London than the North of England for sugary drinks (55.3% vs 49.9%, P=0.035), digital food delivery services (59.0% vs 51.5%, P=0.003) and advertising across transport networks (45.5% vs 28.8%, P<0.001) (see **Supplementary Table S4)**. After adjustments, participants living in London had higher odds of self-reported exposure for sugary drinks (OR 1.27; 95% CI 1.03-1.56), digital food

For peer review only

delivery services (OR 1.39; 95% 1.13-1.71) and advertising across transport networks (OR 2.05; 95% CI 1.65-2.54) compared to those living in the North of England (**Table 2-3**).

Sociodemographic differences in self-reported exposure to advertising for less healthy foods and drinks

The adjusted odds of self-reported exposure to advertising overall were higher in the middle socioeconomic group (C1C2) compared to the high socioeconomic group (AB) (OR 1.48; 95% CI 1.06-2.07) (**Table 2**). When stratified by product category, sociodemographic differences in self-reported exposure to advertising were found in three of the five categories studied (processed HFSS foods, sugary cereals and sweet snacks). Socioeconomic differences were observed for these three product categories, with lower socioeconomic position associated with higher adjusted odds of self-reported exposure (ORs ranging from 1.41 to 1.67 for low compared to high). There was indication of a socioeconomic gradient for all three product categories, although the CIs for the middle and low socioeconomic groups overlapped. Gender differences were also observed for processed HFSS foods, with higher adjusted odds for women compared to men (OR 1.44; 95% CI 1.13-1.84). For sweet snacks, participants who were full-time employees had marginally higher adjusted odds of reporting advertising exposure compared to participants who were not looking for work or unable to work (OR 1.40; 95% CI 1.00-1.97). There were no observed associations between sociodemographic characteristics and self-reported exposure to advertising for fast food or sugary drinks. There were some interactions between sociodemographic characteristics and region on their influence on self-report advertising exposure (see **Supplementary Table S5**). Results stratified by region are presented in **Supplementary Tables S6 and S7** where significant.

For peer review only - http://bmjopen.bmj.com/site/about/guidelines.xhtml

Sociodemographic differences in exposure to advertising for digital food delivery services

Higher adjusted odds of reporting exposure to advertising for digital food delivery services were observed in the lower socioeconomic groups compared to the highest: low (OR 1.47; 95% CI 1.05-2.05) and middle (OR 1.39; 95% CI 1.08-1.80). The adjusted odds were also higher among participants aged 18-34 years (OR 2.08; 95% CI 1.20-3.59), 35-44 years (OR 1.93; 95% CI 1.15-3.36) and 55-64 years (OR 1.53; 95% CI 1.00-2.35) compared to those aged ≥65 years.

Sociodemographic differences in exposure to advertising by advertising setting

Sociodemographic differences in self-reported exposure were found for traditional advertising, digital advertising and advertising across transport networks (**Table 3**). Adjusted odds of self-reported exposure were higher in the lower socioeconomic groups compared to the high socioeconomic group for traditional advertising: low (OR 1.44; 95% CI 1.00-2.08) and middle (OR 1.52; 95% CI 1.15-2.00). Adjusted odds of self-reported exposure to digital advertising were higher in the low compared to high socioeconomic group (OR 1.50; 95% CI 1.06-2.14), and younger age groups compared to ≥65 years: 18-34 years (OR 3.93, 95% CI 2.18-2.07) and 35-44 years (OR 3.06; 95% CI 1.74-5.40). The adjusted odds of reporting exposure to advertising across transport networks were higher among participants aged 18-34 years compared to ≥65 years (OR 1.96; 95% CI 1.11-3.48), and participants who were full-time employees compared to those not looking for work or unable to work (OR 1.50; 95% CI 1.04-2.17).

Association between advertising exposure and BMI

For peer review only - http://bmjopen.bmj.com/site/about/guidelines.xhtml

Overall self-reported exposure to less healthy food and drink advertising was not associated with BMI category (**Table 4**). However, higher adjusted odds of living with obesity were observed among participants who reported exposure to advertising for digital food delivery services (OR 1.40; 95% CI 1.05-1.88), digital advertising (OR 1.80; 95% CI 1.33-2.44) and advertising in recreational environments (OR 1.46; 95% CI 1.02-2.09).

DISCUSSION

Summary of findings

This is one of the few studies to investigate sociodemographic correlates of exposure to less healthy food and drink advertising in UK adults. Overall, exposure was high, with 84.7% of participants reporting seeing or hearing advertising for less healthy foods and drinks and/or digital food delivery services in the past 7 days. Lower socioeconomic position was associated with increased odds of advertising exposure. Overall, the middle (C1C2) socioeconomic group had higher odds of exposure compared to the most affluent group (AB). For three of the five food and drink product categories (processed HFSS foods, sugary cereals and sweet snacks) and digital food delivery services, increased odds of self-reported exposure to advertising were observed for lower socioeconomic groups (DE and C1C2 compared to AB). When stratified by advertising setting, higher odds of reporting exposure among lower socioeconomic groups were observed for traditional (C1C2 and DE) and digital advertising (DE only). Younger participants had higher odds of self-reported exposure to advertising for digital food delivery services compared to participants aged ≥65 years, except those aged 45-54 years. Younger participants were also more likely to report exposure to digital advertising (18-34 and 35-44 age groups) and advertising across transport networks (18-34 age group). Participants who were full-time employees had

higher self-reported odds of exposure to advertising for sweet snacks and advertising across transport networks. Women had higher odds of reporting exposure to advertising for processed HFSS foods than men. Exposure to advertising for digital food delivery services, digital advertising and advertising in recreational environments were associated with obesity.

Strengths & limitations

Whilst most of the existing research on less healthy food and drink advertising focuses on children, this study adds to the evidence for sociodemographic differences in advertising exposure in adults. There are some strengths and limitations of this study that should be noted. There is no standard method for measuring advertising exposure. The questions used in this study were informed by those used in the International Food Policy Study.[12] The survey questions asked about the products advertised and the advertising setting, giving us the opportunity to explore what was advertised and where. This provided a broader perspective relative to studies focusing on one form of advertising.[18,19] The inclusion of digital food delivery services in the survey also provided insight into a fast-growing and understudied channel for purchasing less healthy foods and drinks.

The cross-sectional nature of this study and short recall period may mean that exposure as measured is not representative of typical exposure, for example due to the seasonality of advertising.[46] Furthermore, we did not account for the intensity of exposure, as we only categorised participants as exposed or not, and did not measure how many advertisements participants saw or heard. The cross-sectional nature of this study also limits our ability to establish causality and reverse causation could explain some of the associations observed.

For peer review only - http://bmjopen.bmj.com/site/about/guidelines.xhtml

Self-reported information is subject to misreporting. For example, height is often over-reported and weight under-reported, leading to inaccuracies in BMI.[47] Therefore, our interpretation of the associations found in this study should be cautious. We also only used one proxy measure of socioeconomic position and therefore may not have fully captured socioeconomic variation in advertising exposure. Further studies using objectively measured BMI and advertising exposure, and other proxy measures of socioeconomic position such as educational level, should be conducted to confirm our findings. Other sociodemographic characteristics not available in our study, such as ethnicity, may also be associated with advertising exposure and warrant study in future.

Generalisability

Our study sample was likely representative of populations in London and the North of England, as Kantar assesses representativeness of their panels every four weeks.[42] However, our study sample was not representative of the UK as a whole. Nonetheless, our study sample appears similar to national samples and the general population in terms of distribution of most sociodemographic characteristics. Most participants (71.6%) were female, which is likely due to more women being the main food shopper within a household than men. A similar proportion of main food providers were women (73.3%) in latest wave (2014/15 to 2016/17) of the National Diet and Nutrition Survey (NDNS), which aims to be representative of the UK population.[48] A similar proportion of participants also reported not having children in the household in our sample (71.5%) as in the NDNS (69.8%), but this is higher than in the general population (58.0%).[49] Having children may impact on advertising exposure (e.g. through different advertisements shown around children's

For peer review only - http://bmjopen.bmj.com/site/about/guidelines.xhtml

television programmes) or recall of advertising exposure (e.g. through 'pester power').[50,51] A greater proportion of participants in our sample reported being in work (62.2%) than in the NDNS (56.2%). Discounting those with missing BMIs, the proportion of participants in each BMI category in this study was similar to that found in the Health Survey for England 2018.[21] However, the number of participants in the underweight category was too small for any statistically meaningful interpretations to be drawn for this group in our study.

Interpretation

Socioeconomic differences in self-reported advertising exposure were observed across various product categories and advertising settings, with more disadvantaged groups more likely to report exposure than the least disadvantaged group. These findings are consistent with previous studies that documented socioeconomic differences in exposure to less healthy food and drink advertising in the UK using more objective measures of exposure.[18,19] The alignment between objective and self-reported measures suggests that self-reported measures may be appropriate proxies for advertising exposure. Further, our findings are consistent with those in the wider international literature.[17,52,53]

There are various possible explanations for the associations between sociodemographic characteristics and self-reported advertising exposure observed in this study (**Figure 1**). Low socioeconomic position, younger age, full-time working and being female may be driving higher exposure to less healthy advertising. For example, for socioeconomic position, this could be through a greater concentration of advertising for less healthy products in more disadvantaged areas. Studies in the US have found that advertising for HFSS foods and

For peer review only

drinks was more prevalent in more deprived neighbourhoods,[19] and that the types of products advertised also differ depending on neighbourhood demographics.[54] We did not capture the urbanicity of participants' locations in this study, which may also influence the density and types of outdoor advertising present in the local neighbourhood.[54] Another potential influence on actual advertising exposure is differential use of public transport across population subgroups. Participants who were full-time employees were more likely to report exposure to advertising across transport networks, as were younger participants. These groups may have been more exposed to advertising across transport networks due to more frequent public transport use.[55,56] Differences in advertising exposure could also be associated with known sociodemographic differences in screen time and use of certain media, such as radio and social media.[57,58]

Alternatively, the observed differences may be due to differences in recall rather than actual exposure. The content of advertisements may be designed to appeal to certain population groups resulting in different cognitive responses to advertisements. Identity salience (defined as heightened sensitivity to identity-relevant stimuli) may make advertisements more impactful to certain groups (i.e. advertisements that feel relevant may be more impactful) and therefore more memorable.[59] Some groups may also be more receptive to advertising due to higher cognitive load. One experiment showed greater effect of advertising on food choices when cognitive load was higher.[11] The authors argued that the effect of advertising on food choices could therefore be exacerbated in low socioeconomic groups because of the greater cognitive load of daily pressures. Receptiveness to advertising may also decrease with age.[36] Equally, sociodemographic

For peer review only

differences in reporting may reflect differential social desirability bias across population groups.

Differences in reported exposure to advertising across transport networks between London and the North of England may be partly explained by differences in the extent of public transport infrastructure, with London having the most extensive transport network in the UK.[32] Participants living in London also reported greater exposure to advertising for digital food delivery services. This is may be due to the greater penetration of digital food delivery services in London while these services are continuing to expand.[60]

Self-reported exposure to advertising for digital food delivery services, digital advertising and advertising in recreational environments were independently associated with higher odds of obesity. Whilst it is implausible that advertising exposure over seven days directly caused obesity, advertising exposure may be stable over time and suggestive of a long-term effect of advertising on the consumption of less healthy products. Reverse causality, whereby people living with obesity are more likely to see or hear, recall or report advertising of less healthy products, may also explain the associations observed.

A large majority of participants reported seeing or hearing some form of advertising for less healthy foods and drinks and/or digital food delivery services, indicating high prevalence of exposure across the UK adult population. This level of exposure was consistent with levels found elsewhere using self-reported measures.[12,61] However, advertising can influence consumer behaviour unconsciously.[62] Therefore, self-reported measures may

For peer review only

underestimate advertising exposure. When objectively measured, one study found that children were exposed to HFSS advertising 27.3 times per day.[63]

The variety of advertising strategies used by companies to advertise HFSS products suggests that restrictions may need to be broadened beyond those proposed in the government's plans to tackle obesity.[30,64] Digital food delivery services were disproportionately advertised through digital channels, meaning that exposure to such advertising can be readily accessed from any location. Exposure to fast food advertising was the most commonly reported of the product categories studied and no sociodemographic variation was observed, which may indicate the pervasiveness of fast food advertising in particular.

Conclusions

Exposure to advertising of less healthy foods and drinks was highly prevalent, with adults in lower socioeconomic groups and younger adults more likely to report exposure. Though these groups may have more objective exposure to advertising, this may also be partly due to increased receptiveness to, or recall of, advertising. Future studies should explore associations using objective measures to confirm our findings. Our findings suggest that broad advertising restrictions are likely needed to reduce exposure to HFSS advertising in adults and may help to reduce sociodemographic differences in exposure to less healthy product advertising. Research evaluating the impacts of such policies should be a priority. Longitudinal studies of advertising exposure and consumption of less healthy foods and drinks would help to determine whether the relationship is causal. If so, interventions should be designed to harness the potential for advertising restrictions to reduce advertising exposure in the groups that are most exposed.

For peer review only - http://bmjopen.bmj.com/site/about/guidelines.xhtml

List of abbreviations

BMI	Body Mass Index
CI(s)	Confidence Interval(s)
FMCG	Fast Moving Consumer Goods
HFSS	High fat, salt and sugar
NDNS	National Diet and Nutrition Survey
NRS	National Readership Survey
UK	United Kingdom

DECLARATIONS

Ethics approval and consent to participate

Ethical approval for this study was granted by the London School of Hygiene & Tropical Medicine Research Ethics Committee (Application No. 16297 /RR/11721). Written informed consent was obtained from all participants.

Consent for publication

Not applicable.

Availability of data and materials

Data were collected via an online survey linked to social and demographic data from Kantar WorldPanel Plus. The terms of our data agreement with Kantar mean that we cannot share linked social and demographic data, however access to the online survey data for the

For peer review only

For peer review only - http://bmjopen.bmj.com/site/about/guidelines.xhtml

purposes of replication only may be possible. Please contact the study Principal Investigator, Professor Steven Cummins (steven.cummins@lshtm.ac.uk).

Competing interests

The authors declare that they have no competing interests.

Funding

The National Institute for Health Research (NIHR) School for Public Health Research is a partnership between the Universities of Sheffield; Bristol; Cambridge; Imperial; and University College London; The London School for Hygiene and Tropical Medicine (LSHTM); LiLaC – a collaboration between the Universities of Liverpool and Lancaster; and Fuse - The Centre for Translational Research in Public Health, a collaboration between Newcastle, Durham, Northumbria, Sunderland and Teesside Universities. This study is funded by the NIHR School for Public Health Research (SPHR) (Grant Reference Number PD-SPH-2015). SC is also funded by Health Data Research UK (HDR-UK). HDR-UK is an initiative funded by the UK Research and Innovation, Department of Health and Social Care (England) and the devolved administrations, and leading medical research charities. JA, MW and TB are supported by the MRC Epidemiology Unit, University of Cambridge [grant number MC/UU/12015/6] and Centre for Diet and Activity Research (CEDAR), a UK Clinical Research Collaboration (UKCRC) Public Health Research Centre of Excellence. Funding for CEDAR from the British Heart Foundation, Cancer Research UK, Economic and Social Research Council, Medical Research Council, the National Institute for Health Research [grant numbers ES/G007462/1 and MR/K023187/1], and the Wellcome Trust [grant number 087636/Z/08/Z], under the auspices of the UK Clinical Research Collaboration, is gratefully

For peer review only

acknowledged. CT is funded by the NIHR Applied Research Collaboration East of England. AAL is a member of Fuse, the Centre for Translational Research in Public Health (www.fuse.ac.uk). Fuse is a Public Health Research Centre of Excellence funded by the five North East Universities of Durham, Newcastle, Northumbria, Sunderland and Teesside. FdV is partly funded by NIHR Applied Research Collaboration West (NIHR ARC West) at University Hospitals Bristol NHS Foundation Trust. The views expressed are those of the authors and do not necessarily represent those of any of the above named funders. The funders had no role in the design of the study, or collection, analysis and interpretation of the data, or in the decision to publish, or in writing the manuscript.

Authors' contributions

SC, AY, JA, EB, TB, LC, FdV, ME, VE, AAL, KL, OM, MP, CT and MW developed the research questions. SC, JA, EB, TB, LC, FdV, ME, VE, AAL, KL, OM, MP, CT and MW contributed to the design the survey. AY and SC designed the analysis of the survey data. AY led on the analysis with support from SC. AY led on the writing of the manuscript with support from SC. All authors critically reviewed and edited the manuscript. All authors read and approved the final manuscript.

Acknowledgements

Not applicable.

For peer review only - http://bmjopen.bmj.com/site/about/guidelines.xhtml

REFERENCES

1. World Health Organization. Marketing of foods high in fat, salt and sugar to children: update 2012–2013 [Internet]. 2013. Available from: http://www.euro.who.int/pubrequest

2. HM Government. Introducing further advertising restrictions on TV and online for products high in fat, sugar and salt (HFSS) [Internet]. London; 2019. Available from: https://assets.publishing.service.gov.uk/government/uploads/system/uploads/attachment_data/file/807378/hfss-advertising-consultation-10-april-2019.pdf

3. The Food Foundation. The Broken Plate. London; 2019.

4. The Food Foundation. Veg Facts A briefing by the Food Foundation [Internet]. London; 2016. Available from: https://foodfoundation.org.uk/wp-content/uploads/2016/11/FF-Veg-Doc-V5.pdf

5. Boyland EJ, Nolan S, Kelly B, Tudur-Smith C, Jones A, Halford JCG, et al. Advertising as a cue to consume: A systematic review and meta-analysis of the effects of acute exposure to unhealthy food and nonalcoholic beverage advertising on intake in children and adults. Am J Clin Nutr. American Society for Nutrition; 2016;103:519–33.

6. Sadeghirad B, Duhaney T, Motaghipisheh S, Campbell NRC, Johnston BC. Influence of unhealthy food and beverage marketing on children's dietary intake and preference: a systematic review and meta-analysis of randomized trials. Obes Rev. 2016;17:945–59.

7. Aschemann-Witzel J, Perez-Cueto FJ, Niedzwiedzka B, Verbeke W, Bech-Larsen T. Lessons for public health campaigns from analysing commercial food marketing success factors: A case study. BMC Public Health. 2012;12:139.

8. World Health Organisation. Reducing the impact of marketing of foods and non-alcoholic beverages on children [Internet]. 2019 [cited 2020 Jul 28]. Available from: https://www.who.int/elena/titles/food_marketing_children/en/

9. Harris JL, Bargh JA, Brownell KD. Priming Effects of Television Food Advertising on Eating Behavior. Heal Psychol. 2009;28:404–13.

10. Koordeman R, Anschutz DJ, van Baaren RB, Engels RCME. Exposure to soda commercials affects sugar-sweetened soda consumption in young women. An observational experimental study. Appetite [Internet]. Elsevier Ltd; 2010;54:619–22. Available from: http://dx.doi.org/10.1016/j.appet.2010.03.008

11. Zimmerman FJ, Shimoga S V. The effects of food advertising and cognitive load on food

For peer review only

choices. BMC Public Health. 2014;14:342.

12. Forde H, White M, Levy L, Greaves F, Hammond D, Vanderlee L, et al. The relationship between self-reported exposure to sugar-sweetened beverage promotions and intake: Cross-sectional analysis of the 2017 international food policy study. Nutrients. 2019;11:3047.

13. Critchlow N, Bauld L, Thomas C, Hooper L, Vohra J. Awareness of marketing for high fat, salt, or sugar (HFSS) foods, and the association with higher weekly consumption among adolescents: A rejoinder to the UK Government's consultations on marketing regulation. Public Health Nutr. 2020;23:2637–46.

14. Cairns G. A critical review of evidence on the sociocultural impacts of food marketing and policy implications. Appetite [Internet]. Elsevier; 2019;136:193–207. Available from: https://doi.org/10.1016/j.appet.2019.02.002

15. OECD. Obesity Update 2017 [Internet]. Paris; 2017. Available from: www.oecd.org/health/obesity-update.htm

16. Baker C. House of Commons Library | Obesity Statistics. Obes. Stat. (Briefing Pap. Number 3336). London: House of Commons; 2019.

17. Backholer K, Gupta A, Zorbas C, Bennett R, Huse O, Chung A, et al. Differential exposure to, and potential impact of, unhealthy advertising to children by socio-economic and ethnic groups: A systematic review of the evidence. Obes Rev. 2021;22:e13144.

18. Adams J, Tyrrell R, Adamson AJ, White M. Socio-economic differences in exposure to television food advertisements in the UK: a cross-sectional study of advertisements broadcast in one television region. Public Health Nutr [Internet]. 2011 [cited 2019 Aug 22];15:487–94. Available from: http://www.nrs.co.uk/lifestyle.htm

19. Adams J, Ganiti E, White M. Socio-economic differences in outdoor food advertising in a city in Northern England. Public Health Nutr [Internet]. Cambridge University Press; 2011 [cited 2019 Aug 23];14:945–50. Available from: https://www.cambridge.org/core/product/identifier/S1368980010003332/type/journal_article

20. Steel N, Hardcastle AC, Bachmann MO, Richards SH, Mounce LTA, Clark A, et al. Economic inequalities in burden of illness, diagnosis and treatment of five long-term conditions in England: Panel study. BMJ Open. 2014;4:e005530.

21. NHS Digital. Health Survey for England 2018: Data tables. 2019.

For peer review only - http://bmjopen.bmj.com/site/about/guidelines.xhtml

22. Lewer D, Jayatunga W, Aldridge RW, Edge C, Marmot M, Story A, et al. Premature mortality attributable to socioeconomic inequality in England between 2003 and 2018: an observational study. Lancet Public Heal [Internet]. 2019 [cited 2019 Dec 8];5:PE33-E41. Available from: https://linkinghub.elsevier.com/retrieve/pii/S2468266719302191

23. Chambers SA, Freeman R, Anderson AS, MacGillivray S. Reducing the volume, exposure and negative impacts of advertising for foods high in fat, sugar and salt to children: A systematic review of the evidence from statutory and self-regulatory actions and educational measures. Prev Med (Baltim) [Internet]. Elsevier Inc.; 2015;75:32–43. Available from: http://dx.doi.org/10.1016/j.ypmed.2015.02.011

24. Kovic Y, Noel JK, Ungemack JA, Burleson JA. The impact of junk food marketing regulations on food sales: an ecological study. Obes Rev. 2018;19:761–9.

25. World Health Organization. Evaluating implementation of the WHO set of recommendations on the marketing of foods and non-alcoholic beverages to children: Progress, challenges and guidance for next steps in the WHO European Region [Internet]. Copenhagen; 2018. Available from: http://www.euro.who.int/__data/assets/pdf_file/0003/384015/food-marketing-kids-eng.pdf

26. Pollack CC, Kim J, Emond JA, Brand J, Gilbert-Diamond D, Masterson TD. Prevalence and strategies of energy drink, soda, processed snack, candy and restaurant product marketing on the online streaming platform Twitch. Public Health Nutr. 2020;23:2793–803.

27. Jaworowska A, Blackham T, Stevenson L, Davies IG. Determination of salt content in hot takeaway meals in the United Kingdom. Appetite. 2012;59:517–22.

28. Jaworowska A, Blackham T, Long R, Taylor C, Ashton M, Stevenson L, et al. Nutritional composition of takeaway food in the UK. Nutr Food Sci. 2014;44:414–30.

29. Robinson E, Jones A, Whitelock V, Mead BR, Haynes A. (Over)eating out at major UK restaurant chains: Observational study of energy content of main meals. BMJ. 2018;363:k4982.

30. Montgomery KC, Chester J. Interactive Food and Beverage Marketing: Targeting Adolescents in the Digital Age. J Adolesc Heal [Internet]. Elsevier Ltd; 2009;45:S18–29. Available from: http://dx.doi.org/10.1016/j.jadohealth.2009.04.006

31. OutSmart. Who sees OOH? [Internet]. 2021 [cited 2021 Feb 26]. Available from: https://www.outsmart.org.uk/who-sees-ooh

For peer review only - http://bmjopen.bmj.com/site/about/guidelines.xhtml

32. Transport for London. Advertising Annual Report 2017/18. London; 2018.

33. Tatum M. The Advertising Report. 2017.

34. Advertising Association. Advertising pays 7: UK advertising's digital revolution [Internet]. London; 2019. Available from: https://www.adassoc.org.uk/wp-content/uploads/2019/06/Ad-Pays-7-UK-Advertisings-Digital-Revolution-compressed.pdf

35. Forbes Insights. Reaching the right audience. How brands are using audience targeting in digital advertising. [Internet]. Jersey City; 2015. Available from: https://info.quantcast.com/rs/quantcast/images/Quantcast_F-Insights_Report-36_Final-Web.pdf

36. Freeman B, Kelly B, Vandevijvere S, Baur L. Young adults: Beloved by food and drink marketers and forgotten by public health? Health Promot Int. 2016;31:954–61.

37. Buston O. Lifting the lid on the secretive online strategies global food giants are using to manipulate British children. 2020.

38. Yau A, Adams J, Monsivais P. Time trends in adherence to UK dietary recommendations and associated sociodemographic inequalities, 1986-2012: a repeated cross-sectional analysis. Eur J Clin Nutr [Internet]. 2019 [cited 2019 May 21];73:997–1005. Available from: http://www.nature.com/articles/s41430-018-0347-z

39. Mills SDH, Tanner LM, Adams J. Systematic literature review of the effects of food and drink advertising on food and drink-related behaviour, attitudes and beliefs in adult populations. Obes Rev. 2013;14:303–14.

40. Cummins S. Restricting advertising of high fat, salt and sugar foods and drinks on the Transport for London network: evaluation of a natural experiment [Internet]. 2019. Available from: http://www.isrctn.com/ISRCTN19928803

41. Berger N, Cummins S, Smith RD, Cornelsen L. Recent trends in energy and nutrient content of take-home food and beverage purchases in Great Britain: an analysis of 225 million food and beverage purchases over 6 years. BMJ Nutr Prev Heal. 2019;2:63–71.

42. Cornelsen L, Berger N, Cummins S, Smith RD. Socio-economic patterning of expenditures on 'out-of-home' food and non-alcoholic beverages by product and place of purchase in Britain. Soc Sci Med [Internet]. Elsevier; 2019;235:112361. Available from: https://doi.org/10.1016/j.socscimed.2019.112361

43. National Readership Survey. Social Grade [Internet]. 2018 [cited 2020 Sep 22]. Available from: http://www.nrs.co.uk/nrs-print/lifestyle-and-classification-data/social-grade/

For peer review only

44. Cairns G, Angus K, Hastings G, Caraher M. Systematic reviews of the evidence on the nature, extent and effects of food marketing to children. A retrospective summary. Appetite. 2013.

45. Hammond D. International Food Policy Study [Internet]. 2019. Available from: http://foodpolicystudy.com/about/

46. Adams J, Simpson E, White M. Variations in food and drink advertising in UK monthly women's magazines according to season, magazine type and socio-economic profile of readers: A descriptive study of publications over 12 months. BMC Public Health. 2011;11:368.

47. Ng CD. Biases in self-reported height and weight measurements and their effects on modeling health outcomes. SSM - Popul Heal [Internet]. Elsevier; 2019;7:100405. Available from: https://doi.org/10.1016/j.ssmph.2019.100405

48. NatCen Social Research. MRC Elsie Widdowson Laboratory. National Diet and Nutrition Survey Years 1-9, 2008/09-2016/17. [data collection]. 15th Edition. [data collection]. 15th Edition; 2019.

49. Office for National Statistics. Families and households in the UK: 2019. Southampton: ONS; 2019.

50. Huang CY, Reisch LA, Gwozdz W, Molnár D, Konstabel K, Michels N, et al. Pester power and its consequences: Do European children's food purchasing requests relate to diet and weight outcomes? Public Health Nutr. 2016;19:2393–403.

51. Whalen R, Harrold J, Child S, Halford J, Boyland E. The health halo trend in UK television food advertising viewed by children: The rise of implicit and explicit health messaging in the promotion of unhealthy foods. Int J Environ Res Public Health. 2018;15:560.

52. Kumanyika S, Grier S. Targeting interventions for ethnic minority and low-income populations. Futur Child. 2006;16:187–207.

53. Lucan SC, Maroko AR, Sanon OC, Schechter CB. Unhealthful Food-and-Beverage Advertising in Subway Stations: Targeted Marketing, Vulnerable Groups, Dietary Intake, and Poor Health. J Urban Heal. Journal of Urban Health; 2017;94:220–32.

54. Kelly B, Cretikos M, Rogers K, King L. The commercial food landscape: Outdoor food advertising around primary schools in Australia. Aust N Z J Public Health. 2008;32:522–8.

55. Campaign for Better Transport. Why getting transport right matters to young people [Internet]. London; 2013. Available from:

For peer review only

http://www.bettertransport.org.uk/files/Young_People_and_Buses_FINAL_forweb_0.pdf
56. Transport for London. TfL Bus User Survey 2014. London; 2014.
57. Stamatakis E, Coombs N, Rowlands A, Shelton N, Hillsdon M. Objectively-assessed and self-reported sedentary time in relation to multiple socioeconomic status indicators among adults in England: A cross-sectional study. BMJ Open. 2014;4:e006034.
58. Ofcom. Adults' media use and attitudes report [Internet]. London; 2019. Available from: http://stakeholders.ofcom.org.uk/market-data-research/media-literacy/media-lit-research/adults-2013/
59. Forehand MR, Deshpandé R, Reed A. Identity salience and the influence of differential activation of the social self-schema on advertising response. J Appl Psychol. 2002;87:1086–99.
60. Just Eat Group. 2018 Capital Markets Day. London; 2018.
61. Cancer Research UK. 86% of Teens Exposed to Junk Food Ads on Social Media [Internet]. 2020 [cited 2021 Feb 26]. Available from: https://www.cancerresearchuk.org/about-us/cancer-news/press-release/2020-12-18-86-of-teens-exposed-to-junk-food-ads-on-social-media
62. Folkvord F, Anschütz DJ, Boyland E, Kelly B, Buijzen M. Food advertising and eating behavior in children. Curr Opin Behav Sci. 2016;9:26–31.
63. Signal LN, Stanley J, Smith M, Barr MB, Chambers TJ, Zhou J, et al. Children's everyday exposure to food marketing: An objective analysis using wearable cameras. Int J Behav Nutr Phys Act. International Journal of Behavioral Nutrition and Physical Activity; 2017;14:137.
64. Department of Health and Social Care: Global Public Health Directorate: Obesity Food and Nutrition / 10800. Childhood obesity: a plan for action Chapter 2 [Internet]. London: HM Government; 2018 [cited 2018 Aug 2]. Available from: www.nationalarchives.gov.uk/doc/open-government-licence/

For peer review only

For peer review only - http://bmjopen.bmj.com/site/about/guidelines.xhtml

FIGURE CAPTIONS

Figure 1. Potential explanations for sociodemographic differences in exposure to advertising for less healthy foods and drinks

For peer review only

For peer review only - http://bmjopen.bmj.com/site/about/guidelines.xhtml

Table 1. Sociodemographic characteristics of study population (*n*=1552)

Sociodemographic characteristic		Total, N (%)	London, N (%)	North, N (%)	X^2 (P-Value)
Sex	Male	441 (28.4)	213 (30.2)	228 (26.9)	2.05 (0.152)
	Female	1,111 (71.6)	492 (69.8)	619 (73.1)	
Age group, years	18-34	188 (12.1)	65 (9.20)	123 (14.5)	18.73 (0.001)***
	35-44	299 (19.3)	160 (22.7)	139 (16.4)	
	45-54	411 (26.5)	196 (27.8)	215 (25.4)	
	55-64	335 (21.6)	145 (20.6)	190 (22.4)	
	≥65	319 (20.6)	139 (19.7)	180 (21.3)	
Socioeconomic position	AB	341 (22.0)	177 (25.1)	164 (19.4)	7.92 (0.019)*
	C1C2	926 (59.7)	409 (58.0)	517 (61.0)	
	DE	285 (18.4)	119 (16.9)	166 (19.6)	
Children in the household	No	1,110 (71.5)	507 (71.9)	603 (71.2)	0.10 (0.754)
	Yes	442 (28.5)	198 (28.1)	244 (28.8)	
Working status	Full-time	612 (39.4)	285 (40.4)	327 (36.8)	19.88 (0.001)***
	Part-time	223 (14.4)	91 (12.9)	132 (15.6)	
	Self-employed	131 (8.4)	78 (11.6)	53 (6.3)	
	Retired	342 (22.0)	145 (20.6)	197 (23.3)	
	Not looking/ unable to work	214 (13.8)	87 (12.3)	127 (15.0)	
	Other	30 (1.9)	19 (2.7)	11 (1.3)	
BMI	Underweight	31 (2.0)	21 (3.0)	10 (1.2)	11.91 (0.018)*
	Normal	479 (30.9)	236 (33.5)	243 (28.7)	
	Overweight	425 (27.4)	180 (25.5)	245 (28.9)	
	Obese	336 (21.7)	143 (20.3)	193 (22.8)	
	Missing	281 (18.1)	125 (17.7)	156 (18.4)	
Region	North	847 (54.6)	N/A	N/A	N/A
	London	705 (45.4)			

For peer review only

Table 2. Sociodemographic correlates of advertising exposure stratified by product/service advertised (*n*=1552)

Sociodemographic characteristic		Any	Processed HFSS foods	Sugary drinks	Sugary cereals	Sweet snacks	Fast food	Digital food delivery services
Sex	Male	REF	REF	REF	REF	REF	REF	REF
	Female	1.11 (0.81-1.53)	1.44 (1.13-1.84)**	1.09 (0.86-1.37)	0.89 (0.70-1.12)	1.16 (0.92-1.46)	1.02 (0.79-1.31)	0.93 (0.74-1.18)
Age group	18-34	1.11 (0.52-2.35)	1.21 (0.68-2.17)	1.07 (0.62-1.83)	1.12 (0.65-1.94)	0.99 (0.57-1.70)	1.24 (0.68-2.24)	2.08 (1.20-3.59)**
	35-44	1.21 (0.59-2.50)	1.39 (0.80-2.44)	1.09 (0.65-1.83)	1.41 (0.83-2.39)	0.96 (0.57-1.61)	1.29 (0.73-2.29)	1.93 (1.15-3.26)**
	45-54	1.09 (0.56-2.12)	0.97 (0.58-1.61)	0.96 (0.59-1.54)	1.18 (0.73-1.92)	0.86 (0.54-1.39)	1.25 (0.74-2.12)	1.40 (0.87-2.26)
	55-64	1.05 (0.58-1.90)	1.16 (0.73-1.84)	1.21 (0.79-1.85)	1.39 (0.90-2.14)	1.06 (0.69-1.62)	1.42 (0.88-2.28)	1.53 (1.00-2.35)*
	≥65	REF	REF	REF	REF	REF	REF	REF
Socioeconomic position	AB	REF	REF	REF	REF	REF	REF	REF
	C1C2	1.48 (1.06-2.07)*	1.42 (1.09-1.84)**	1.22 (0.95-1.57)	1.50 (1.16-1.95)**	1.31 (1.02-1.68)*	1.25 (0.95-1.64)	1.39 (1.08-1.80)**
	DE	1.30 (0.84-2.02)	1.67 (1.17-2.38)**	1.36 (0.97-1.88)	1.54 (1.10-2.16)**	1.41 (1.01-1.96)*	1.28 (0.90-1.84)	1.47 (1.05-2.05)*
Children in the household	No	REF	REF	REF	REF	REF	REF	REF
	Yes	0.72 (0.50-1.04)	0.78 (0.59-1.05)	0.82 (0.63-1.08)	1.07 (0.81-1.40)	0.91 (0.69-1.20)	0.79 (0.59-1.06)	0.77 (0.59-1.02)
Working status	Full-time	1.05 (0.66-1.66)	1.13 (0.78-1.63)	1.17 (0.81-1.59)	1.17 (0.83-1.65)	1.40 (1.00-1.97)*	1.12 (0.77-1.63)	1.22 (0.87-1.72)
	Part-time	1.00 (0.60-1.69)	0.96 (0.64-1.45)	0.97 (0.66-1.42)	1.01 (0.69-1.50)	1.04 (0.71-1.53)	0.96 (0.63-1.46)	0.93 (0.63-1.37)
	Self-employed	1.13 (0.60-2.12)	0.80 (0.50-1.29)	0.88 (0.56-1.38)	0.89 (0.56-1.40)	1.09 (0.70-1.70)	1.18 (0.72-1.95)	0.84 (0.54-1.32)
	Retired	1.06 (0.53-2.11)	1.24 (0.72-2.13)	1.06 (0.64-1.73)	1.33 (0.81-2.20)	1.19 (0.72-1.96)	1.13 (0.65-1.96)	1.16 (0.70-1.91)
	Not looking/ unable to work	REF	REF	REF	REF	REF	REF	REF
	Other	1.13 (0.37-3.49)	0.80 (0.36-1.80)	1.18 (0.54-2.57)	1.27 (0.58-2.76)	1.30 (0.60-2.84)	0.70 (0.31-1.57)	1.00 (0.45-2.18)
Region	London	REF	REF	REF	REF	REF	REF	REF
	North	1.26 (0.95-1.68)	1.10 (0.88-1.37)	1.27 (1.03-1.56)*	1.05 (0.86-1.29)	0.91 (0.74-1.12)	1.09 (0.87-1.36)	1.39 (1.13-1.71)**

Models adjusted for sex, age group, social class, children in the household, working status and region
* P<0.05, ** P<0.01, *** P<0.001

For peer review only - http://bmjopen.bmj.com/site/about/guidelines.xhtml

Table 3. Sociodemographic correlates of advertising exposure stratified by advertising setting (*n*=1552)

Sociodemographic characteristic		Traditional	Digital	Recreational	Functional	Transport
Sex	Male	REF	REF	REF	REF	REF
	Female	1.19 (0.92-1.54)	0.80 (0.63-1.02)	0.94 (0.71-1.26)	0.96 (0.76-1.21)	0.89 (0.70-1.14)
Age group	18-34	0.64 (0.34-1.18)	3.93 (2.18-7.08)***	1.37 (0.70-2.70)	1.27 (0.74-2.18)	1.96 (1.11-3.48)*
	35-44	0.74 (0.41-1.34)	3.06 (1.74-5.40)***	1.27 (0.66-2.44)	1.12 (0.67-1.88)	1.50 (0.87-2.61)
	45-54	0.77 (0.44-1.34)	1.69 (0.99-2.87)	0.84 (0.46-1.55)	0.89 (0.56-1.43)	0.99 (0.59-1.65)
	55-64	0.94 (0.57-1.54)	1.38 (0.85-2.24)	1.13 (0.66-1.94)	0.99 (0.65-1.51)	0.94 (0.59-1.49)
	≥65	REF	REF	REF	REF	REF
Socioeconomic position	AB	REF	REF	REF	REF	REF
	C1C2	1.52 (1.15-2.00)**	1.22 (0.93-1.60)	1.01 (0.73-1.39)	1.16 (0.90-1.49)	1.08 (0.82-1.41)
	DE	1.44 (1.00-2.08)*	1.50 (1.06-2.14)*	1.04 (0.68-1.59)	1.18 (0.85-1.65)	1.12 (0.78-1.59)
Children in the household	No	REF	REF	REF	REF	REF
	Yes	0.93 (0.69-1.26)	0.96 (0.72-1.27)	0.74 (0.52-1.05)	0.88 (0.67-1.15)	0.79 (0.60-1.06)
Working status	Full-time	1.01 (0.68-1.48)	1.28 (0.90-1.82)	1.18 (0.76-1.82)	1.07 (0.76-1.49)	1.50 (1.04-2.17)*
	Part-time	0.85 (0.55-1.31)	1.08 (0.73-1.61)	1.05 (0.64-1.73)	0.84 (0.57-1.23)	1.12 (0.74-1.71)
	Self-employed	0.80 (0.49-1.33)	0.93 (0.58-1.48)	0.87 (0.48-1.58)	0.95 (0.61-1.48)	1.55 (0.97-2.49)
	Retired	0.90 (0.50-1.60)	0.84 (0.49-1.45)	0.93 (0.49-1.76)	0.80 (0.49-1.32)	1.02 (0.59-1.76)
	Not looking/unable to work	REF	REF	REF	REF	REF
	Other	0.76 (0.32-1.78)	0.58 (0.25-1.36)	1.61 (0.66-3.94)	0.85 (0.39-1.84)	2.18 (0.98-4.86)
Region	London	REF	REF	REF	REF	REF
	North	1.13 (0.90-1.43)	1.20 (0.97-1.49)	1.11 (0.86-1.45)	1.16 (0.94-1.42)	2.05 (1.65-2.54)***

Models adjusted for sex, age group, social class, children in the household, working status and region
* P<0.05, ** P<0.01, *** P<0.001

For peer review only

Table 4. Weight status by exposure to advertising for less healthy foods and drinks (*n*=1552)

**Type of advertising**		**Underweight,** OR (95% CI)	**Overweight,** OR (95% CI)	**Obesity,** OR (95% CI)	**Missing,** OR (95% CI)
Any advertising exposure		0.94 (0.35-2.57)	1.08 (0.75-1.57)	1.08 (0.72-1.60)	0.94 (0.63-1.41)
By product category	Processed HFSS foods	1.31 (0.56-3.04)	1.17 (0.87-1.56)	1.12 (0.82-1.53)	0.89 (0.65-1.21)
	Sugary drinks	1.07 (0.51-2.24)	1.09 (0.84-1.43)	1.00 (0.75-1.33)	1.04 (0.78-1.41)
	Sugary cereals	1.03 (0.49-2.20)	1.21 (0.92-1.59)	1.29 (0.97-1.72)	1.03 (0.76-1.40)
	Sweet snacks	1.14 (0.54-2.39)	1.03 (0.79-1.35)	1.11 (0.83-1.47)	0.79 (0.58-1.06)
	Fast food	0.69 (0.32-1.47)	1.18 (0.88-1.58)	1.31 (0.95-1.80)	1.00 (0.72-1.38)
Digital food delivery services		0.71 (0.33-1.49)	1.22 (0.93-1.60)	1.40 (1.05-1.88)*	1.05 (0.78-1.43)
By advertising setting	Traditional	0.88 (0.40-1.94)	1.32 (0.98-1.79)	1.28 (0.93-1.77)	1.24 (0.89-1.74)
	Digital	1.12 (0.51-2.47)	1.28 (0.96-1.71)	1.80 (1.33-2.44)***	1.12 (0.82-1.54)
	Recreational	1.05 (0.41-2.66)	1.15 (0.81-1.63)	1.46 (1.02-2.09)*	1.07 (0.72-1.58)
	Functional	0.65 (0.31-1.38)	1.01 (0.78-1.32)	1.15 (0.86-1.52)	1.06 (0.79-1.43)
	Transport	0.63 (0.28-1.41)	1.07 (0.80-1.42)	1.20 (0.89-1.63)	0.78 (0.57-1.08)

Model adjusted for sex, age group, social class, children in the household, working status and region
* P<0.05, ** P<0.01, *** P<0.001

For peer review only

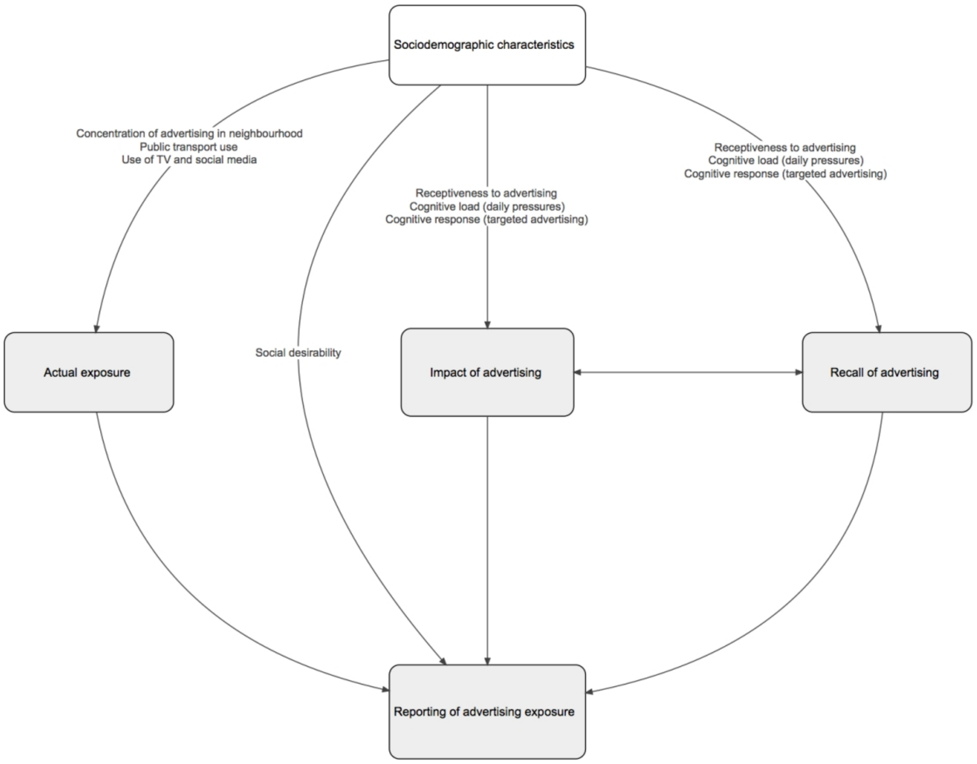

Potential explanations for sociodemographic differences in exposure to advertising for less healthy foods and drinks

525x414mm (72 x 72 DPI)

SUPPLEMENTARY MATERIAL

Supplementary Table S1. Definitions of product categories as presented in survey questions

**Product category**	**Definition in survey**
Processed HFSS foods	Processed foods high in salt, sugar and fat are those such as sugary drinks, meals from fast food chains, ready meals, sit down meals, sugary breakfast cereals, sweet snacks (e.g. chocolate bars, sweets, cookies/biscuits), savoury snacks (e.g. crisps, salted/flavoured nuts) and desserts (cakes, ice-cream and flavoured yoghurts).
Sugary drinks	SUGARY DRINKS are drinks that contain added sugar, like fizzy drinks, fruit drinks, sports drinks, energy drinks, diluted cordials/squash, chocolate milk, and specialty flavoured hot drinks that have added sugar but DO NOT INCLUDE pure fruit juice.
Sugary cereals	SUGARY CEREALS are those that contain added sugar such as chocolate or other flavouring/ fillings e.g. Coco Pops, Krave or are sweetened with frosting or added sugar e.g. Frosties, Sugar Puffs. DO NOT INCLUDE cereals like Weetabix, Ready Brek, Oatmeal/Porridge).
Sweet snacks	SWEET SNACKS are foods such as chocolate bars, cookies/biscuits, danish pastries, granola bars, sweets or other confectionary.
Fast food	These can be MEALS, FOODS OR DRINKS from fast-food chains or take-aways (e.g., Burger King, Dominos, McDonalds, KFC, fish and chip shops, Indian or Chinese Takeaway).

For peer review only

Supplementary Table S2. Number (%) of participants who reported seeing or hearing advertising for less healthy products and digital food delivery services in the past 7 days by sociodemographic characteristics, stratified by HFSS product category (*n*=1552)

Sociodemographic characteristic		Any	Processed HFSS foods	Sugary drinks	Sugary cereals	Sweet snacks	Fast food	Digital food delivery services
All		1314 (84.7)	1059 (68.2)	813 (52.4)	653 (42.1)	853 (55.0)	1107 (71.3)	852 (54.9)
Sex, N (%)	Male	370 (83.9)	277 (62.8)	228 (51.7)	195 (44.2)	235 (53.3)	316 (71.7)	249 (56.5)
	Female	944 (85.0)	782 (70.4)	585 (52.7)	458 (41.2)	618 (55.6)	791 (71.2)	603 (54.3)
X^2 (P-value)		0.28 (0.598)	8.36 (0.004)**	0.11 (0.734)	1.16 (0.281)	0.70 (0.404)	0.03 (0.857)	0.61 (0.435)
Age group (years), N (%)	18-34	157 (83.5)	129 (68.6)	96 (51.1)	74 (36.4)	105 (55.9)	131 (69.7)	113 (60.1)
	35-44	253 (84.6)	211 (70.6)	155(51.8)	134 (44.8)	161 (53.9)	210 (70.2)	176 (58.9)
	45-54	348 (84.7)	263 (64.0)	206 (50.1)	166 (40.4)	216 (52.6)	294 (71.5)	220 (53.5)
	55-64	286 (85.4)	235 (70.2)	190 (56.7)	151 (45.1)	193 (57.6)	252 (75.2)	191 (57.0)
	≥65	270 (84.6)	221 (69.3)	166 (52.0)	128 (40.1)	178 (55.8)	220 (69.0)	152 (47.7)
X^2 (P-value)		0.32 (0.988)	4.91 (0.297)	3.55 (0.471)	3.70 (0.448)	2.21 (0.697)	3.79 (0.435)	11.6 (0.020)*
Socioeconomic position, N (%)	AB	276 (80.9)	210 (61.6)	165 (48.4)	119 (34.9)	169 (49.6)	231 (67.7)	168 (49.3)
	C1C2	798 (86.2)	643 (69.4)	492 (53.1)	410 (44.3)	523 (56.5)	671 (72.5)	525 (56.7)
	DE	240 (84.2)	206 (72.3)	156 (54.7)	124 (43.5)	161 (56.5)	205 (71.9)	159 (55.8)
X^2 (P-value)		5.32 (0.070)	9.73 (0.008)*	3.02 (0.220)	9.29 (0.010)*	5.15 (0.076)	2.78 (0.249)	5.67 (0.059)
Children in the household, N (%)	No	951 (85.7)	768 (69.2)	598 (53.9)	465 (41.9)	621 (56.0)	807 (72.7)	615 (55.4)
	Yes	363 (82.1)	291 (65.8)	215 (48.6)	188 (42.5)	232 (52.5)	300 (67.9)	237 (53.6)
X^2 (P-value)		3.07 (0.080)	1.64 (0.200)	3.47 (0.063)	0.05 (0.817)	1.53 (0.217)	3.61 (0.058)	0.41 (0.523)
Working status, N (%)	Full-time	519 (84.8)	418 (68.3)	328 (53.6)	267 (43.6)	350 (57.2)	445 (72.7)	365 (59.6)
	Part-time	187 (83.9)	151 (67.7)	112 (50.2)	91 (40.8)	116 (52.0)	154 (69.1)	115 (51.6)
	Self-employed	113 (86.3)	82 (62.6)	64 (48.9)	49 (37.4)	68 (51.9)	97 (74.1)	67 (51.2)
	Retired	290 (84.8)	241 (70.5)	181 (52.9)	145 (42.4)	192 (56.1)	241 (70.5)	171 (50.0)
	Not looking/ unable to work	179 (83.6)	148 (69.2)	111 (51.9)	87 (40.7)	110 (51.4)	151 (70.6)	117 (54.7)
	Other	26 (86.7)	19 (63.3)	17 (56.7)	14 (46.7)	17 (56.7)	19 (63.3)	17 (56.7)
X^2 (P-value)		0.65 (0.986)	3.15 (0.676)	1.71 (0.887)	2.38 (0.795)	3.82 (0.575)	2.73 (0.741)	10.66 (0.059)
Region, N (%)	London	706 (83.4)	485 (68.8)	390 (55.3)	300 (42.6)	377 (53.5)	509 (72.2)	416 (59.0)
	North	608 (86.2)	574 (67.8)	423 (49.9)	353 (41.7)	476 (56.2)	598 (70.6)	436 (51.5)
X^2 (P-value)		2.47 (0.116)	0.19 (0.666)	4.46 (0.035)*	0.12 (0.728)	1.15 (0.283)	0.48 (0.489)	8.81 (0.003)**
* P<0.05, ** P<0.01, *** P<0.001								

For peer review only

Supplementary Table S3. Number (%) of participants who reported seeing or hearing advertising for less healthy products and digital food delivery services in the past 7 days by sociodemographic characteristics, stratified by advertising setting (*n*=1552)

Sociodemographic characteristic		Traditional	Digital	Recreational	Functional	Transport
All		1148 (74.0)	600 (38.7)	292 (18.8)	799 (51.5)	565 (36.4)
Sex, N (%)	Male	318 (72.1)	179 (40.6)	87 (19.7)	231 (52.4)	172 (39.0)
	Female	830 (74.7)	421 (37.9)	205 (18.5)	568 (51.1)	393 (35.4)
X^2 (P-value)		1.11 (0.293)	0.97 (0.325)	0.34 (0.562)	0.20 (0.655)	1.80 (0.180)
Age group (years), N (%)	18-34	130 (69.2)	108 (57.5)	41 (21.8)	107 (56.9)	87 (46.3)
	35-44	215 (71.9)	155 (51.8)	61 (20.4)	161 (53.9)	128 (42.8)
	45-54	300 (73.0)	157 (38.2)	66 (16.1)	204 (49.6)	144 (35.0)
	55-64	258 (77.0)	109 (32.5)	69 (20.6)	173 (51.6)	109 (32.5)
	≥65	245 (76.8)	71 (22.3)	55 (17.2)	154 (48.3)	97 (30.4)
X^2 (P-value)		6.08 (0.193)	91.4 (<0.001)***	4.85 (0.303)	4.77 (0.312)	20.66 (<0.001)***
Socioeconomic position, N (%)	AB	231 (67.7)	121 (35.5)	64 (18.8)	167 (49.0)	125 (36.7)
	C1C2	703 (75.9)	363 (39.2)	175 (18.9)	484 (52.3)	342 (36.9)
	DE	214 (75.1)	116 (40.7)	53 (18.6)	148 (51.9)	98 (34.4)
X^2 (P-value)		8.88 (0.012)**	2.07 (0.356)	0.01 (0.993)	1.11 (0.574)	0.62 (0.732)
Children in the household, N (%)	No	834 (75.1)	391 (35.2)	217 (19.6)	573 (51.6)	401 (36.1)
	Yes	314 (71.0)	209 (47.3)	75 (17.0)	226 (51.1)	164 (37.1)
X^2 (P-value)		2.75 (0.097)	19.39 (<0.001)***	1.38 (0.240)	0.03 (0.862)	0.13 (0.718)
Working status, N (%)	Full-time	451 (73.7)	284 (46.4)	126 (20.6)	333 (54.4)	254 (41.5)
	Part-time	161 (72.2)	91 (40.8)	40 (17.9)	107 (48.0)	72 (32.3)
	Self-employed	93 (71.0)	50 (38.2)	21 (16.0)	68 (51.9)	57 (43.5)
	Retired	262 (76.6)	78 (22.8)	59 (17.3)	163 (47.7)	100 (29.2)
	Not looking/ unable to work	160 (74.8)	88 (41.1)	38 (17.8)	113 (52.8)	66 (30.8)
	Other	21 (70.0)	9 (30.0)	8 (26.7)	15 (50.0)	16 (53.3)
X^2 (P-value)		2.54 (0.770)	53.67 (<0.001)***	3.95 (0.556)	5.38 (0.371)	25.52 (<0.001)***
Region, N (%)	London	528 (75.9)	287 (40.7)	139 (19.7)	376 (53.3)	321 (45.5)
	North	620 (73.2)	313 (37.0)	153 (18.1)	423 (49.9)	244 (28.8)
X^2 (P-value)		0.57 (0.449)	2.29 (0.130)	0.69 (0.407)	1.77 (0.183)	46.48 (<0.001)***
* P<0.05, ** P<0.01, *** P<0.001						

For peer review only

Supplementary Table S4. Number (%) of participants who reported seeing or hearing advertising for less healthy products and digital food delivery services in the past 7 days by product/service advertised and advertising setting, stratified by region (*n*=1552)

**Advertised product/service**	**Region**	All	Traditional	Digital	Recreational	Functional	Transport
All, N (%)	All	1314 (84.7)	1148 (74.0)	600 (38.7)	292 (18.8)	799 (51.5)	565 (36.4)
	London	608 (86.2)	528 (74.9)	287 (40.7)	139 (19.7)	376 (53.3)	321 (45.5)
	North	706 (83.4)	620 (73.2)	313 (37.0)	153 (18.1)	423 (49.9)	244 (28.8)
X^2 (P-value)		2.47 (0.116)	0.57 (0.449)	2.29 (0.130)	0.69 (0.407)	1.77 (0.183)	46.48 (<0.001)***
Processed HFSS foods, N (%)	All	1059 (68.2)	853 (55.0)	384 (24.7)	135 (8.7)	547 (35.2)	376 (24.2)
	London	485 (68.8)	387 (54.9)	177 (25.1)	59 (8.4)	253 (35.9)	223 (31.6)
	North	574 (67.8)	466 (55.0)	207 (24.4)	76 (9.0)	294 (34.7)	153 (18.1)
X^2 (P-value)		0.19 (0.666)	0.002 (0.961)	0.09 (0.762)	0.18 (0.674)	0.23 (0.629)	38.58 (<0.001)***
Sugary drinks, N (%)	All	813 (52.4)	596 (38.3)	223 (14.4)	114 (7.4)	318 (20.5)	206 (13.3)
	London	390 (55.3)	279 (39.6)	105 (14.9)	56 (7.9)	155 (22.0)	117 (16.6)
	North	423 (49.9)	316 (37.3)	118 (13.9)	58 (6.9)	163 (19.2)	89 (10.5)
X^2 (P-value)		4.46 (0.035)*	0.84 (0.361)	0.29 (0.591)	0.68 (0.410)	1.77 (0.183)	12.39 (<0.001)***
Sugary cereals, N (%)	All	653 (42.1)	502 (32.4)	123 (7.9)	48 (3.1)	215 (13.9)	75 (4.8)
	London	300 (42.6)	230 (32.6)	62 (8.8)	26 (3.7)	100 (14.2)	56 (7.9)
	North	353 (41.7)	272 (32.1)	61 (7.2)	22 (2.6)	115 (13.6)	19 (2.2)
X^2 (P-value)		0.12 (0.728)	0.05 (0.830)	1.33 (0.248)	1.53 (0.217)	0.12 (0.730)	27.18 (<0.001)***
Sweet snacks, N (%)	All	853 (55.0)	639 (41.2)	218 (14.1)	101 (6.5)	339 (21.8)	171 (11.0)
	London	377 (53.5)	273 (38.7)	101 (14.3)	45 (6.4)	163 (23.1)	109 (15.5)
	North	476 (56.2)	366 (43.2)	117 (13.8)	56 (6.6)	176 (20.8)	62 (7.3)
X^2 (P-value)		1.15 (0.283)	3.20 (0.074)	0.08 (0.772)	0.03 (0.856)	1.24 (0.266)	26.01 (<0.001)***
Fast food, N (%)	All	1107 (71.3)	855 (55.1)	312 (20.1)	103 (6.6)	380 (24.5)	327 (21.1)
	London	509 (72.2)	386 (54.8)	137 (19.4)	53 (7.5)	179 (25.4)	198 (28.1)
	North	598 (70.6)	469 (55.4)	175 (20.7)	50 (5.9)	201 (23.7)	129 (15.2)
X^2 (P-value)		0.48 (0.489)	0.06 (0.807)	0.36 (0.548)	1.62 (0.203)	0.57 (0.449)	38.23 (<0.001)***
Digital food delivery services, N (%)	All	852 (54.9)	647 (41.7)	297 (19.1)	37 (2.4)	115 (7.4)	126 (8.1)
	London	416 (59.0)	299 (42.4)	163 (23.1)	34 (3.4)	66 (9.4)	96 (13.6)
	North	436 (51.5)	348 (41.1)	134 (15.8)	13 (1.5)	49 (5.8)	30 (3.5)
X^2 (P-value)		8.81 (0.003)**	0.28 (0.598)	13.25 (<0.001)**	5.78 (0.016)*	7.17 (0.007)*	52.36 (<0.001)***
* P<0.05, ** P<0.01, *** P<0.001							

For peer review only

Supplementary Table S5. Interaction terms (P-values) for interactions between sociodemographic characteristics and region on self-reported advertising exposure

Advertising type	Sex##Region	Age group##Region	Socioeconomic position##Region	Children##Region	Working status##Region
Any advertising	1.52 (0.677)	3.91 (0.917)	5.81 (0.325)	4.23 (0.238)	2.83 (0.993)
Processed HFSS foods	10.64 (0.014)**	7.35 (0.600)	9.64 (0.086)	3.41 (0.332)	6.15 (0.863)
Sugary drinks	2.31 (0.511)	6.40 (0.700)	6.62 (0.251)	3.45 (0.327)	4.59 (0.949)
Sugary cereal	1.96 (0.580)	5.76 (0.764)	13.08 (0.023)*	2.32 (0.508)	14.30 (0.217)
Sweet snacks	2.60 (0.457)	3.83 (0.922)	8.88 (0.114)	1.47 (0.690)	9.19 (0.605)
Fast food	0.08 (0.995)	4.05 (0.908)	3.37 (0.643)	3.48 (0.323)	4.28 (0.961)
Digital food delivery services	4.27 (0.233)	16.41 (0.059)	8.56 (0.128)	4.15 (0.245)	13.14 (0.284)
Traditional advertising	2.26 (0.520)	7.82 (0.553)	11.37 (0.044)*	1.39 (0.709)	9.29 (0.595)
Digital advertising	5.79 (0.122)	47.08 (<0.001)***	9.82 (0.081)	3.27 (0.352)	22.98 (0.018)*
Advertising in recreational environments	2.32 (0.508)	10.03 (0.348)	3.90 (0.564)	5.17 (0.160)	5.90 (0.880)
Advertising in functional environments	1.60 (0.660)	9.93 (0.357)	3.81 (0.577)	3.20 (0.362)	9.51 (0.575)
Advertising across transport networks	5.31 (0.151)	29.90 (<0.001)***	7.43 (0.190)	7.25 (0.065)	16.69 (0.117)
* P<0.05, ** P<0.01, *** P<0.001					

For peer review only

Supplementary Table S6. Sociodemographic correlates of advertising exposure stratified by product/service advertised and region where interactions were significant (*n*=1552)

Product/service advertised	Sex	Age group	Socioeconomic position	Children in the household	Working status
Any advertising	No interaction	No interaction	No interaction	No interaction	No interaction
Processed HFSS foods	London<br>1.18 (0.82-1.70)<br>North of England<br>1.70 (1.22-2.37)**	No interaction	No interaction	No interaction	No interaction
Sugary drinks	No interaction	No interaction	No interaction	No interaction	No interaction
Sugary cereal	No interaction	No interaction	London<br>C1C2: 1.35 (0.94-1.95)<br>DE: 1.75 (1.06-2.88)*<br>North of England<br>C1C2: 1.62 (1.11-2.36)*<br>DE: 1.41 (0.88-2.25)	No interaction	No interaction
Sweet snacks	No interaction	No interaction	No interaction	No interaction	No interaction
Fast food	No interaction	No interaction	No interaction	No interaction	No interaction
Digital food delivery services	No interaction	No interaction	No interaction	No interaction	No interaction

For peer review only

Supplementary Table S7. Sociodemographic correlates of advertising exposure stratified by advertising setting and region where interactions were significant (*n*=1552)

Advertising setting	Sex	Age group	Socioeconomic position	Children in the household	Working status
Traditional	No interaction	No interaction	London<br>C1C2: 1.41 (0.95-2.10)<br>DE: 1.90 (1.06-3.41)*<br>North of England<br>C1C2: 1.53 (1.04-2.25)*<br>DE: 1.17 (0.72-1.91)	No interaction	No interaction
Digital	No interaction	London<br>35-44: 0.84 (0.45-1.55)<br>45-54: 0.37 (0.20-0.68)**<br>55-64: 0.27 (0.14-0.52)***<br>≥65: 0.15 (0.06-0.37)***<br>North of England<br>35-44: 0.72 (0.44-1.18)<br>45-54: 0.46 (0.28-0.74)**<br>55-64: 0.42 (0.25-0.71)***<br>≥65: 0.37 (0.17-0.82)***	No interaction	No interaction	London<br>Part-time: 0.69 (0.40-1.16)<br>Self-employed: 0.69 (0.40-1.17)<br>Retired: 0.64 (0.31-1.35)<br>At home: 0.50 (0.29-0.88)*<br>Other: 0.08 (0.02-0.37)**<br>North of England<br>Part-time: 0.99 (0.64-1.55)<br>Self-employed: 0.71 (0.38-1.33)<br>Retired: 0.67 (0.34-1.32)<br>At home: 1.06 (0.67-1.66)<br>Other: 2.67 (0.75-9.55)
Recreational	No interaction	No interaction	No interaction	No interaction	No interaction
Functional	No interaction	No interaction	No interaction	No interaction	No interaction
Transport	No interaction	London<br>35-44: 0.72 (0.39-1.30)<br>45-54: 0.52 (0.29-0.93)*<br>55-64: 0.55 (0.29-1.04)*<br>≥65: 0.73 (0.32-1.68)<br>North of England<br>35-44: 0.86 (0.52-1.43)<br>45-54: 0.49 (0.30-0.82)**<br>55-64: 0.44 (0.25-0.77)**<br>≥65: 0.34 (0.15-0.78)**	No interaction	No interaction	No interaction

For peer review only

STROBE Statement—checklist of items that should be included in reports of observational studies

	Item No.	Recommendation	Page No.	Relevant text from manuscript
**Title and abstract**	1	(*a*) Indicate the study's design with a commonly used term in the title or the abstract	1	Cross-sectional analysis
		(*b*) Provide in the abstract an informative and balanced summary of what was done and what was found	2-3	
**Introduction**				
Background/rationale	2	Explain the scientific background and rationale for the investigation being reported	5-6	
Objectives	3	State specific objectives, including any prespecified hypotheses	7	
**Methods**				
Study design	4	Present key elements of study design early in the paper	7-11	
Setting	5	Describe the setting, locations, and relevant dates, including periods of recruitment, exposure, follow-up, and data collection	7-11	
Participants	6	(*a*) *Cohort study*—Give the eligibility criteria, and the sources and methods of selection of participants. Describe methods of follow-up<br>*Case-control study*—Give the eligibility criteria, and the sources and methods of case ascertainment and control selection. Give the rationale for the choice of cases and controls<br>*Cross-sectional study*—Give the eligibility criteria, and the sources and methods of selection of participants	7	
		(*b*) *Cohort study*—For matched studies, give matching criteria and number of exposed and unexposed<br>*Case-control study*—For matched studies, give matching criteria and the number of controls per case	N/A	
Variables	7	Clearly define all outcomes, exposures, predictors, potential confounders, and effect modifiers. Give diagnostic criteria, if applicable	8-10	
Data sources/ measurement	8*	For each variable of interest, give sources of data and details of methods of assessment (measurement). Describe comparability of assessment methods if there is more than one group	*8-10*	
Bias	9	Describe any efforts to address potential sources of bias	N/A	
Study size	10	Explain how the study size was arrived at	7	

Continued on next page

For peer review only - http://bmjopen.bmj.com/site/about/guidelines.xhtml

Quantitative variables	11	Explain how quantitative variables were handled in the analyses. If applicable, describe which groupings were chosen and why	8-10
Statistical methods	12	(*a*) Describe all statistical methods, including those used to control for confounding	11
		(*b*) Describe any methods used to examine subgroups and interactions	11
		(*c*) Explain how missing data were addressed	8-11
		(*d*) *Cohort study*—If applicable, explain how loss to follow-up was addressed<br>*Case-control study*—If applicable, explain how matching of cases and controls was addressed<br>*Cross-sectional study*—If applicable, describe analytical methods taking account of sampling strategy	N/A
		(*e*) Describe any sensitivity analyses	N/A
**Results**			
Participants	13*	(a) Report numbers of individuals at each stage of study—eg numbers potentially eligible, examined for eligibility, confirmed eligible, included in the study, completing follow-up, and analysed	11
		(b) Give reasons for non-participation at each stage	10
		(c) Consider use of a flow diagram	
Descriptive data	14*	(a) Give characteristics of study participants (eg demographic, clinical, social) and information on exposures and potential confounders	11
		(b) Indicate number of participants with missing data for each variable of interest	10
		(c) *Cohort study*—Summarise follow-up time (eg, average and total amount)	
Outcome data	15*	*Cohort study*—Report numbers of outcome events or summary measures over time	
		Case-control study—Report numbers in each exposure category, or summary measures of exposure	
		Cross-sectional study—Report numbers of outcome events or summary measures	*11*
Main results	16	(*a*) Give unadjusted estimates and, if applicable, confounder-adjusted estimates and their precision (eg, 95% confidence interval). Make clear which confounders were adjusted for and why they were included	12-15
		(*b*) Report category boundaries when continuous variables were categorized	8-11
		(*c*) If relevant, consider translating estimates of relative risk into absolute risk for a meaningful time period	

Continued on next page

For peer review only - http://bmjopen.bmj.com/site/about/guidelines.xhtml

Other analyses	17	Report other analyses done—eg analyses of subgroups and interactions, and sensitivity analyses	
**Discussion**			
Key results	18	Summarise key results with reference to study objectives	15-16
Limitations	19	Discuss limitations of the study, taking into account sources of potential bias or imprecision. Discuss both direction and magnitude of any potential bias	16-17
Interpretation	20	Give a cautious overall interpretation of results considering objectives, limitations, multiplicity of analyses, results from similar studies, and other relevant evidence	17-20
Generalisability	21	Discuss the generalisability (external validity) of the study results	17
**Other information**			
Funding	22	Give the source of funding and the role of the funders for the present study and, if applicable, for the original study on which the present article is based	22-23

*Give information separately for cases and controls in case-control studies and, if applicable, for exposed and unexposed groups in cohort and cross-sectional studies.

Note: An Explanation and Elaboration article discusses each checklist item and gives methodological background and published examples of transparent reporting. The STROBE checklist is best used in conjunction with this article (freely available on the Web sites of PLoS Medicine at http://www.plosmedicine.org/, Annals of Internal Medicine at http://www.annals.org/, and Epidemiology at http://www.epidem.com/). Information on the STROBE Initiative is available at www.strobe-statement.org.

For peer review only
